# Identification of MRAP protein family as broad‐spectrum GPCR modulators

**DOI:** 10.1002/ctm2.1091

**Published:** 2022-10-31

**Authors:** Meng Wang, Xiaozhu Wang, Bopei Jiang, Yue Zhai, Jihong Zheng, Liu Yang, Xiaolu Tai, Yunpeng Li, Shaliu Fu, Jing Xu, Xiaowei Lei, Zhe Kuang, Cong Zhang, Xuanxuan Bai, Mingyu Li, Tao Zan, Shen Qu, Qingfeng Li, Chao Zhang

**Affiliations:** ^1^ Department of Plastic and Reconstructive Surgery Shanghai Ninth People's Hospital Shanghai Jiao Tong University School of Medicine Shanghai China; ^2^ School of Life Sciences and Technology Tongji University Shanghai China; ^3^ Department of Endocrinology and Metabolism National Metabolic Management Center Shanghai Tenth People's Hospital School of Medicine Tongji University Shanghai China; ^4^ Fujian Provincial Key Laboratory of Innovative Drug Target Research School of Pharmaceutical Sciences Xiamen University Xiamen China

**Keywords:** bulk RNA‐seq, energy homeostasis, GPCR, MRAP1, MRAP2, single‐cell RNA‐seq

## Abstract

**Background:**

The melanocortin receptor accessory proteins (MRAP1 and MRAP2) are well‐known endocrine regulators for the trafficking and signalling of all five melanocortin receptors (MC1R–MC5R). The observation of MRAP2 on regulating several non‐melanocortin G protein‐coupled receptors (GPCRs) has been sporadically reported, whereas other endogenous GPCR partners of the MRAP protein family are largely unknown.

**Methods:**

Here, we performed single‐cell transcriptome analysis and drew a fine GPCR blueprint and MRAPs‐associated network of two major endocrine organs, the hypothalamus and adrenal gland at single‐cell resolution. We also integrated multiple bulk RNA‐seq profiles and single‐cell datasets of human and mouse tissues, and narrowed down a list of 48 GPCRs with strong endogenous co‐expression correlation with MRAPs.

**Results:**

36 and 46 metabolic‐related GPCRs were consequently identified as novel interacting partners of MRAP1 or MRAP2, respectively. MRAPs exhibited protein–protein interactions and varying pharmacological properties on the surface translocation, constitutive activities and ligand‐stimulated downstream signalling of these GPCRs. Knockdown of MRAP2 expression by hypothalamic administration of adeno‐associated virus (AAV) packed shRNA stimulated body weight gain in mouse model. Co‐injection of corticotropinreleasing factor (CRF), the agonist of corticotropin releasing hormone receptor 1 (CRHR1), suppressed feeding behaviour in a MRAP2‐dependent manner.

**Conclusions:**

Collectively, our study has comprehensively elucidated the complex GPCR networks in two major endocrine organs and redefined the MRAP protein family as broad‐spectrum GPCR modulators. MRAP proteins not only serve as a vital endocrine pivot on the regulation of global GPCR activities in vivo that could explain the composite physiological phenotypes of the MRAP2 null murine model but also provide us with new insights of the phenotyping investigation of GPCR–MRAP functional complexes.

## Graphical abstract

1. We performed a single‐cell transcriptome analysis of adrenal gland and hypothalamus and drew a fine endogenous blueprint of all the emerging GPCRs and MRAP proteins.
2. 36 and 46 metabolic‐related GPCRs were consequently identified as novel interacting partners of MRAP1 or MRAP2, respectively.
3. Our study has comprehensively elucidated the complex GPCR networks in two major endocrine organs and redefined the MRAP protein family as broad‐spectrum GPCR modulators.

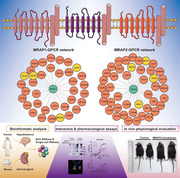

## 1 INTRODUCTION

G protein‐coupled receptor (GPCR) family is the largest family of membrane receptors in the human, which regulates diverse biological functions and serves as one of the most important targets for drug discovery. The melanocortin receptors (MCRs) are a subfamily of the rhodopsin class of GPCRs. Their endogenous accessory protein family named melanocortin receptor accessory protein (MRAP) contains two members in mammals, MRAP1 and MRAP2. They are single‐transmembrane proteins and function as unusual antiparallel homo‐ and heterodimers.^1^, ^2^, ^3^ MRAP1 is required for the plasma membrane trafficking, ligand binding and Gs coupled downstream signalling of melanocortin‐2 receptor (MC2R) in the adrenal gland.^2^, ^4^, ^5^ Loss‐of‐function mutations in MRAP1 clinically caused familial glucocorticoid deficiency type 2.^5^ MRAP2 functions as an essential modulator of MC4R activity in the arcuate nucleus of the hypothalamus.^6^ The global deletion of MRAP2 in mice and loss‐of‐function variants in humans developed severe obesity syndrome.^7^

Subsequently, numerous studies have found the wide distribution of MRAP1 and MRAP2 in several tissues beyond MC2R and MC4R expression, respectively.^7^, ^8^, ^9^ Besides the adrenal gland, knockdown of MRAP1 has been shown to substantially inhibit lipolysis triggered by adrenocor ticotropic hormone (ACTH) in murine adipocytes.^10^ On the other hand, although MC4R and MRAP2 knockout mice developed obese phenotypes, significant differences existed between the two lines.^7^, ^11^ MRAP2‐deficient mice show weight gain but no detectable changes in food intake or energy expenditure,^7^ suggesting the role of MRAP proteins, or at least of MRAP2, was not limited to regulate MCRs. Recently, MRAP2 was reported to modulate the trafficking and signalling of several non‐melanocortin GPCRs, such as prokineticin receptor 1 (PKR1) and orexin receptor 1 (OX1R).^12^, ^13^, ^14^, ^15^ MRAP2 deficiency blocked Ghrelin‐mediated increases in food intake.^15^ Rouault et al.^16^ found that MRAP2 facilitated gastrin‐mediated biased signalling downstream of GHSR1a activation by inhibiting β‐arrestin recruitment to receptors and enhancing Gαq/11‐dependent signalling. Moreover, MRAP2 was expressed in islet δ cells, and both global and δ cell‐targeted deletion of MRAP2 abrogated the pro‐insulin effects of gastrin, identifying MRAP2 as a regulator of insulin secretion.^17^ However, other metabolic‐associated GPCR partners modulated by MRAP1 or MRAP2 have not yet been fully explored.

In this study, in order to comprehensively assess the role of the MRAP protein family on regulating the whole GPCR family, we performed a single‐cell transcriptome analysis of adrenal gland and hypothalamus, the two organs in which expression of MRAP1 and MRAP2 were enriched. We also collected and integrated multiple bulk RNA‐seq and other single‐cell datasets of human and mouse tissues and drew a fine endogenous blueprint of all the emerging GPCRs and MRAP proteins. Consistent with our in silico anticipation, in vitro studies confirmed the protein interaction of these GPCRs with MRAP proteins. Pharmacological analysis further revealed their regulatory roles on the plasma membrane translocation, constitutive activities and ligand‐stimulated intracellular signalling of GPCRs in the presence of various amount of MRAP1 and MRAP2 proteins. Moreover, knockdown of MRAP2 expression by hypothalamic administration of AAV packed shRNA stimulated body weight gain in mouse model. We selected a validated target of MRAP2, the CRHR1 for in vivo physiological evaluation. MRAP2 could inhibit the pharmacological activation of CRHR1 in vitro and hypothalamic co‐injection of CRF, the endogenous agonist of CRHR1 suppressed feeding behaviour in MRAP2 knockdown background. Overall, our data support the idea that MRAP1 and MRAP2 are functioning as broad‐spectrum GPCR modulators and may regulate the energy homeostasis of mammals in an integrated and composite manner.

## 2 MATERIALS AND METHODS

### 2.1 Animals

All C57BL/6 mice were purchased from the GemPharmatech. The mice received food and water ad libitum and were maintained on a regular 12‐h diurnal cycle, and housed in disposable ventilated cages with no more than five animals per cage at 23 ± 2°C. All animal‐related experimental procedures in this study were approved by the Animal Care and Use Committee of Shanghai Jiao Tong University School of Medicine.

### 2.2 Constructs and reagents

The MRAPs (MRAP1 and MRAP2) and 48 selected GPCRs were cloned from a wild‐type mouse cDNA library. The N‐terminal 3xHA and 2xFLAG tag were fused to GPCRs and MRAPs by PCR and the tagged cds sequence of receptor genes were all cloned into a pcDNA3.1(+) vector. Forty‐eight selected GPCRs were tagged with non‐fluorescent fragments of YFP F1 at C‐terminus. The C‐end of MRAP1 and MRAP2 were tagged with FLAG and non‐fluorescent fragments of YFP F2. Primer sequences are available on request. The validity of all constructs in this study was verified by DNA sequencing.

Prostaglandin E2, the agonist of PTGER2 and PTGER4, was purchased from TargetMol. CL‐316,243 (the agonist of ADRB3), L‐161,982 (the antagonist of PTGER4), SR 59230A (the antagonist of ADRB3), PF‐04418948 (the antagonist of Ptger2) and Antalarmin (the antagonist of CRHR1) were purchased from R&D SYSTEMS. CRF, the agonist of CRHR1, was purchased from MCE (MedChemExpress).

### 2.3 GPCR expression analysis in bulk RNA sequencing database and functional enrichment analysis

We employed the online bulk RNA‐seq database GTEx to explore the expression profiles of MRAP/MRAP2 and GPCRs across 22 human tissues (https://www.genome.gov/27543770/gtex‐progress‐update). The overall expression patterns of the detected GPCRs were visualized by a cross‐tissue expression heatmap using R package ComplexHeatmap.^18^ The tissue‐specific enrichment analysis was conducted to identify a specific set of GPCRs in a certain tissue by R package TissueEnrich.^19^ Similarly, we also analysed a published mouse bulk RNA‐se database to investigate the expression patterns of Mrap/Mrap2 and GPCRs across 17 mouse tissues.^20^ The Gene Ontology enrichment analysis on biological process, molecular function and cellular components was performed with GPCRs to identify their involved functional pathways using R package clusterProfiler.^21^

### 2.4 scRNA sequencing of mouse hypothalamus and adrenal glands

A mixture of male and female mouse hypothalamus was utilised for 10× Genomics transcriptome sequencing with mouse brain protocol for cell dissociation.^22^ In brief, the hypothalamus was dissected and immediately chilled on ice upon euthanasia and transferred to enzyme‐digestion fluid (Papain, DNase I, Glutamax). Mouse adrenal glands were collected and dissected in PBS containing 1 mg/ml collagenase/dispase (Roche). The single‐cell digestion process of mouse adrenal gland refers to the methods in the previously published study.^23^ For the scRNA‐seq experiments, 6‐weeks C57BL/6 mouse hypothalamus tissues (two male, two female) or adrenal gland tissues (two male, two female) were collected, and specimens from the same gender were mixed for digestion. After proper dissociation, cells were loaded into a Chromium Single Cell Library & Gel Beads kit v3 i7 Multiplex kit (10× Genomics). The prepared library was sequenced using Illumina Nova 6000 sequencing platform by GENEWIZ company.

### 2.5 Bioinformatics analysis of scRNA sequencing data of the mouse hypothalamus and adrenal gland

The raw reads were first aligned to the mouse genome (mm10) using the 10 × Genomics CellRanger (version 3.0) package with the default parameter settings. The low‐quality cells were filtered out if they met the following criteria: (1) the detected gene number in a cell was below 300 or above 6000; (2) the total counting reads of a cell is below 500 or above 20,000; (3) the percentage of mitochondrial genes was above 25% for hypothalamus and 30% for adrenal gland; (4) the percentage of ribosomal genes was above 25% and 20% for adrenal gland. The high‐quality cells were kept for the subsequent analysis. The count normalisation, PCA dimensional reduction, cell clustering and visualisation steps were conducted with Seurat package.^24^ In brief, the raw counts were normalised and scaled by SCTransform function, followed by the dimensional reduction of PCA analysis with RunPCA function. The cell neighbouring and clustering steps were performed using FindNeighbors and FindClusters functions. The final visualisation was generated by RunTSNE function. To determine the cell identities in the mouse hypothalamus or adrenal gland, we first identified the marker genes in each cell cluster using FindAllMarkers function, and then classic cell markers were used for cell type annotation. The paired‐wise Pearson Correlation Coefficient Analysis of expressed GPCRs in certain cell types was conducted using R package corrr (https://cran.r‐project.org/web/packages/corrr/index.html).

### 2.6 Integration analysis of published hypothalamic and adrenal scRNA sequencing datasets of the human and mouse

The published scRNA‐seq datasets of mouse hypothalamus in four previous studies were obtained with the NCBI Gene Expression Omnibus (GEO) numbers: GSE87544, GSE130597, GSE74672, and GSE125065.^25^ The published scRNA‐seq datasets of human and mouse adrenal gland were obtained from previous studies with GEO numbers of GSM3943047 and GSM3980123 for human, GSM4409674, GSM4409676 and GSM4914026 for mouse.^26^, ^27^ The poor‐quality cells with low coverage of genes and high percentage of mitochondrial and ribosomal genes were removed before the subsequent analysis. As these datasets were generated from different resources, we performed the CCA integrative analysis to remove the potential batch effects using IntegrateData function in Seurat.^24^ The standard Seurat scRNA‐seq pipeline of dimensional reduction, clustering and visualisation was conducted as described above. Similarly, we also analysed the human faetal hypothalamic scRNA‐seq dataset with a NCBI GEO number of GSE118487.^28^ We visualised the expression profiles of MRAPs and GPCRs in different cell types of human and mouse hypothalamus using R packages Seurat^24^ and ComplexHeatmap.^18^

### 2.7 Cell culture and transfection

HEK293T cells were maintained in DMEM/High Glucose (Gibco) supplemented with FBS (10%) and penicillin/streptomycin (1%). Cells were cultured in a humidified atmosphere consisting of 5% CO_2_ at 37°C. Transient transfections were carried out using PEI (Polyscience) according to the manufacturer's instructions. Total plasmid concentrations of all transfections were kept consistent in each experiment by adding empty vectors.

### 2.8 Western blotting and co‐IP

For Western blotting, proteins were extracted from mouse hypothalamus or HEK293T cells and quantified using BCA Kit (Beyotime). Blots were incubated with the following antibodies: anti‐MRAP2 (Thermo Scientific) at 1:1000 dilutions, anti‐actin (Abcam) at 1:4000 dilution, anti‐ERK1/2(Abcam) at 1:1000 dilution, anti‐pERK1/2 (Abcam) at 1:1000 dilution.

For Co‐IP experiments, HEK293T cells were transfected with indicated plasmids and lysed. Lysates were centrifuged and supernatants were incubated with rabbit anti‐HA (Cell Signaling) or rabbit anti‐Flag (Cell Signaling) at 1:5000 dilution overnight at 4°C. Next day, samples were then incubated with Protein A/G Agarose beads (Beyotime) at 4°C for 4 h. Beads were washed three times and resuspended in LDS loading buffer and boiled for 15 min. Proteins were then resolved by SDS/PAGE and detected by mouse anti‐HA (Abclonal) or mouse anti‐Flag (Abclonal) for GPCRs and MRAPs co‐IP experiments. Each IP group was repeated at least three times (*n* = 3).

### 2.9 Bimolecular fluorescence complementation assay

Cells were seeded on poly‐l‐lysine‐coated coverslips of 12‐well plates and transfected with GPCRs and MRAP1/MRAP2‐Flag‐F2. After 24 h transfection, cells were fixed with 4% PFA for 20 min at room temperature. Samples were then incubated with anti‐FLAG antibody (Cell Signaling) at 1:5000 ratio to detect surface expression of MRAPs. After washing three times, samples were incubated with Alexa Fluor594 antibody (Abcam) at 1:5000 ratio. Finally, cells were sealed with ProLong (R) Gold Antifade containing DAPI Molecular Probes (Cell Signaling). Images were captured under 60× oil objective with Zeiss confocal microscopy (LSM880). Each YFP group was repeated at least twice, with three fields of view selected.

### 2.10 ELISA

Cells were cultured in poly‐l‐lysine‐coated 12‐well plates and transfected transiently with the GPCRs (which interacted with MRAPs) and MRAP1 or MRAP2 (1:0, 1:3 and 1:6 ratio of receptor to MRAP1 or MRAP2), or empty vector only. After 24 h transfection, cells were washed three times with PBS, then fixed with 4% polyformaldehyde for 20 min. After fixation, cells were washed three times with PBS and then blocked with 5% milk in PBS for 30 min. Samples were then incubated with 1:2000 mouse anti‐HA antibody for 2 h and 1:2000 anti‐mouse‐HRP antibody (Abclonal) for 1.5 h at RT before the cells were washed three times with PBS. Next, TMB Substrate Solution for ELISA (Beyotime) was added and the reaction was final stopped with equal amount of 2 M sulphuric acid. The absorbance value was measured at 450 nm using a Spectramax I3 plate reader. Three replicate wells were set up for each experimental condition, and the average value was calculated at the time of measurement.

### 2.11 cAMP (CRE‐Luc assays)

Cells were seeded in 24‐well plates and transfected with several plasmids, including CRE‐luciferase reporter plasmid to drive firefly luciferase by a CRE, Renilla luciferase reporter plasmid, along with responding GPCR plasmid (PTGER2, PTGER4, CRHR1 and ADRB3) and either an empty vector or MRAP1/MRAP2 (1:3, 1:6 ratio receptor to MRAP1/MRAP2). After 24 h transfection, cells were incubated with varying concentrations of peptide agonist in DMEM/High Glucose with .1% bovine serum albumin. After 4 h, the medium was removed and Dual‐Glo luciferase buffer with the substrate (Promega) in DMEM/high glucose (1:1) was added. Finally, the luminescence was detected by a Spectramax I3 plate reader (Molecular Devices, Sunnyvale, CA). Three replicate wells were set up for each experimental condition, and the average value was calculated at the time of measurement.

### 2.12 Antagonist competition binding assay

Cells plated in 24‐well plates were transfected the next day with the plasmids listed above. After 24 h of transfection, Antalarmin (antagonist of CRHR1) ranged from 10^−11^ to 10^−6^ M with the presence of 1 nM CRF; SR59230A (antagonist of ADRB3) ranged from 10^−12^ to 10^−7^ M with the presence of 1 nM CL316,243; PF‐044 18948 (antagonist of PTGER2) ranged from 10^−10^ to 10^−5^ M with the presence of 1 nM PEG2; L‐161982 (antagonist of PTGER4) ranged from 10^−9^ to 10^−5^ M with the presence of 0.1 nM PEG2 were added into plates, respectively. After 4 h incubation at 37°C, the cAMP levels were examined by the Dual‐Glo Luciferase Assay kit (Promega) according to the manufacturer's instructions. Three replicate wells were set up for each experimental condition, and the average value was calculated at the time of measurement.

### 2.13 Constitutive activity measurement

For Gs/Gi‐mediated signalling, we transfected with the indicated GPCR plasmids, MRAP1 or MRAP2, CRE‐luciferase reporter plasmid and Renilla luciferase reporter vectors into HEK293 cells per well via PEI reagent. For Gq‐mediated signalling, we transfected with a NFAT luciferase reporter plasmid instead of the plasmid encoding the CRE‐luciferase reporter to detect the calcium flux released by the activation of the Gq signalling pathway. After 24 h transfection, the luminance of cAMP levels or Ca^2 +^ flux was measured by the Dual‐Glo Luciferase Assay kit (Promega). Finally, we used Firefly luciferase/Renilla luciferase to express the constitutive activity of each group of measurements.

### 2.14 Stereotaxic injection

The pscAAV‐U6‐shRNA‐CMV‐EGFP‐tWPA [AAV‐ctrl] and pscAAV‐U6‐shRNA(Mrap2)‐CMV‐EGFP‐tWPA [AAV‐Mrap2] virus (Obio Technology (Shanghai) Corp., Ltd) were injected bilaterally into the hypothalamus of C57BL/6J mice. Eight weeks old mice were anaesthetised with tribromoethanol. Coordinates used for hypothalamus injection were as follows: bregma: anterior–posterior −1.5 mm; lateral ± 0.35 mm; and dorsal–ventral −5.8 mm). The AAVs were infused at a slow rate of 40 nl/min (∼2 × 1012 viral particles/ml) and the injector remained in place for an additional 5 min before.

### 2.15 Drug administration, assessment of food consumption, body weight gain and blood glucose

At least 10–14 days before the experiments, mice were continuously recorded of the amount of consumed food, weight gain and blood glucose every 2 days. Then, mice were received 0.3 μg/μl ICV injection of CRF or 1 μl PBS injection to test the inhibitory effect of the peptide on 2‐h cumulative food intake. Weight gain and blood glucose were recorded every day.

### 2.16 Statistical analysis

Data were analysed using GraphPad Prism 6 program (Graph Pad software, San Diego, CA, USA) and shown as mean ± SEM. To determine the statistically significant differences between groups or time points, two‐sided Student's *t*‐test and one‐way analysis of variance were used. *p < .05, **p < .01, ***p < .001, ****p < .0001.

## 3 RESULTS

### 3.1 Bulk RNA‐seq analysis of the expression profiles of MRAPs and GPCRs across the human and mouse tissues

**FIGURE 1:**
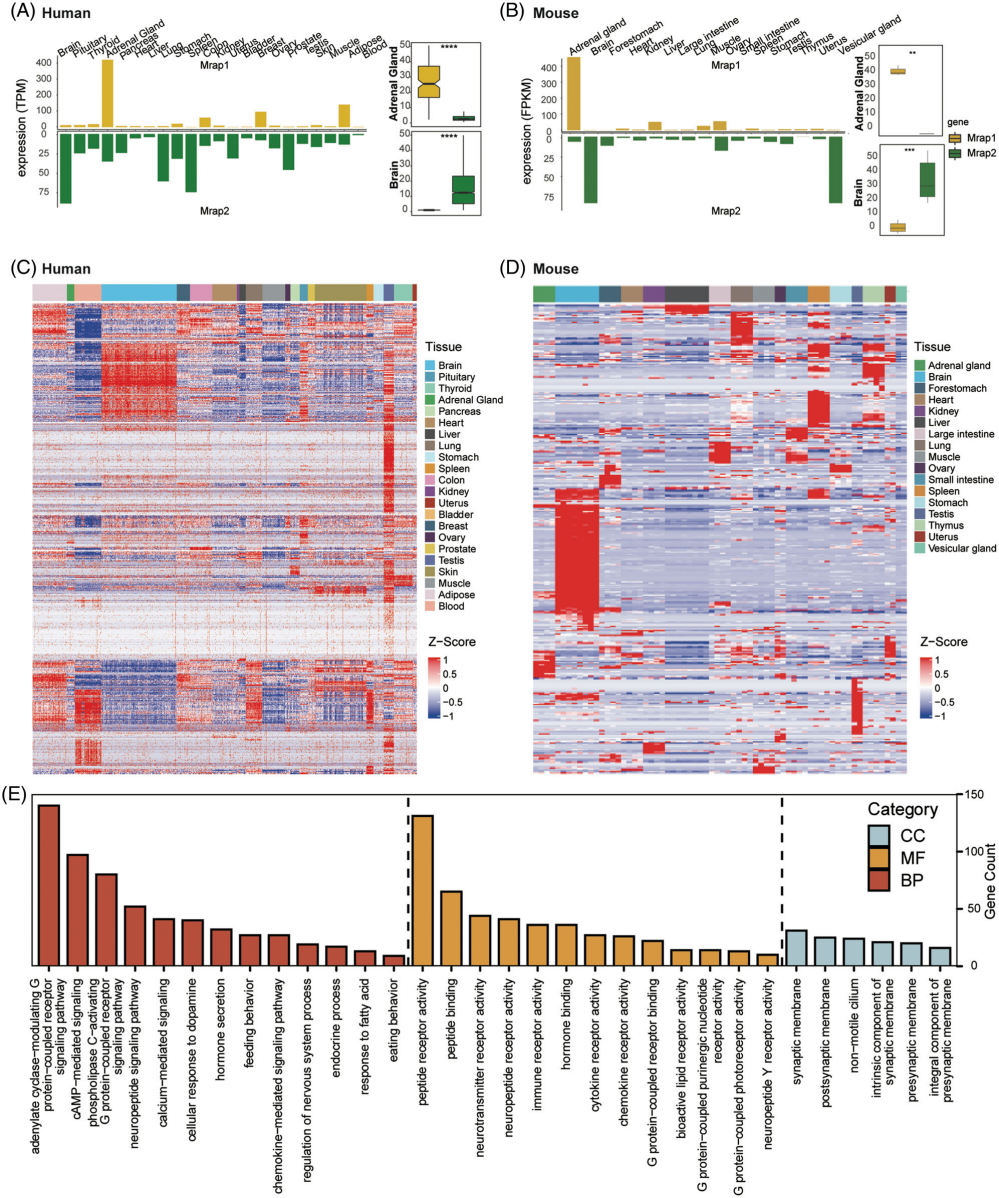
Expression profiles of MRAPs and GPCRs across various human tissues. (A) Expressional analysis of *MRAP1* and *MRAP2* level of the bulk RNA‐seq data across human tissues. Right panel: Comparison of expression levels of *MRAP1* and *MRAP2* in the bulk RNA‐seq data of human brain and adrenal gland. *MRAP1* and *MRAP2* are marked in yellow and green, respectively. (B) Expressional analysis of *MRAP1* and *MRAP2* level of the bulk RNA‐seq data across mouse tissues. Right panel: Comparison of expression levels of *MRAP1* and *MRAP2* in the bulk RNA‐seq data of human brain and adrenal gland. *MRAP1* and *MRAP2* are marked in yellow and green, respectively. (C) Expressional heatmap of all GPCRs in the bulk RNA‐seq datasets across human tissues. (D) Expressional heatmap of all GPCRs in the bulk RNA‐seq datasets across mouse tissues. (E) GO functional enrichment analysis of all GPCRs. The enriched functional pathways with FDR P‐value < .05 were included. CC, cell component; MF, molecular function; BP, biological process.

To assess the expressional network of MRAPs and GPCRs across human and mouse organs and tissues, we performed a tissue‐wide expression analysis of these genes from multiple sets of public bulk RNA‐seq GTEx database. We first evaluated the expression level of human *MRAP1* and *MRAP2* among the 22 human tissues and 17 mouse tissues (Figures 1A and B). In human, *MRAP1* is highly expressed in the adrenal gland. *MRAP2* expresses more widely compared with *MRAP1* with a high abundance in central nervous system (Figure 1A). Additionally, we checked the protein expression of MRAPs in tissues across the body from the Human Protein Atlas database. As shown in Figure S1A, only the protein expression of *MRAP1* is found and mainly enriched in adrenal gland.

We further examined the expression of 823 human GPCRs in the 22 human tissues, and 504 mouse GPCRs in the 17 mouse tissues. The expression levels of the detected GPCR in the different human and mouse tissues were scaled and visualized in Figures 1C and 1D, respectively. The results indicated that the detected GPCRs clearly grouped into clusters in several tissues, especially in brain, adipose, blood, and testis, suggesting their co‐expression patterns in the specific tissues (Figures 1C and D). For instance, we identified a set of brain‐specific GPCRs in human, including ADGRA1, ADGRB1, ADGRB2, ADORA1, CASR, CELSR3, CHRM2, CXCR3, FZD9, GPR12, GPR141, GPR15, GPR22, GPR37L1, GPR61, GPR83, GPR87, GRM2, GRM3, GRM4, HTR2C, HTR5A, MC2R, NPFFR1, OR10J1, OR2H1, OR2L13, P2RY10 and P2RY12 (Figure S1B). Similarly, we also identified a set of adrenal‐specific GPCRs in human, including ADGRA1, ADGRV1, AVPR1A, CHRM2, CYSLTR2, DRD3, DRD5, GPR101, GPR148, GPR22, HCAR3, HTR1D, HTR1E, HTR2B, HTR2C, HTR5A, MC4R, MCHR2, MRAP, NTSR2, OPN5, OR52D1, OR6B3, SSTR4 and TAS2R1 (Figure S1C).

We identified that MRAPs, especially *MRAP2*, are co‐expressed broadly with GPCRs in some human tissues, suggesting potential correlations in exerting their physiological functions. Therefore, we next carried out the functional enrichment analysis to reveal the involved functional pathways of these genes (Figures 1E and S1D and E). Our results showed that these genes were involved in hormone regulation, appetite control and signalling cascades of neuropeptide and G protein receptors (including cAMP activation and calcium influx).

### 3.2 Co‐expression of GPCRs and Mrap2 in both mouse and human hypothalamus

**FIGURE 2:**
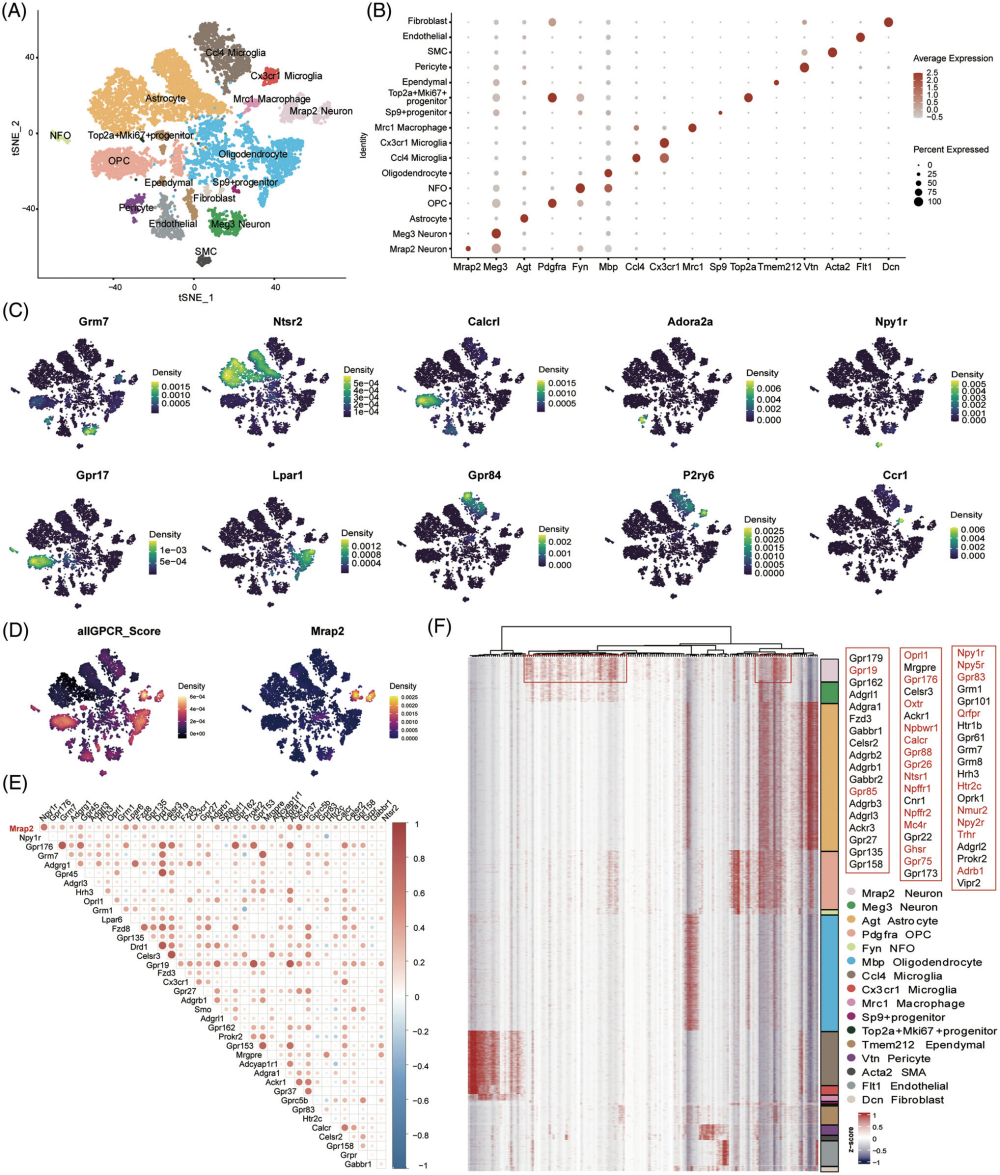
Co‐expression of GPCRs and MRAP2 in different cell types of mouse hypothalamus. (A) tSNE plot of 16 cell types identified in 6‐week WT mouse hypothalamus. (B) Dot plot of classic marker genes used to identify cell identities in mouse hypothalamus. (C) Density plot revealed expression patterns of GPCRs across cell types. (D) Expressions of all the detected GPCRs and Mrap2 across cell types of the mouse hypothalamus. (E) Gene co‐expression correlation networks of GPCRs in Mrap2 neurons. Red colour represents positive correlation, blue colour represents negative correlation. MRAP2 is marked in bold red. Only GPCRs highly correlated with Mrap2 and their absolute correlation coefficients greater than 0.1 were shown. (F) Expression heatmap of the detected GPCRs in mouse hypothalamus. The highly expressed GPCRs in MRAP2 neurons are highlighted in the red box.

The hypothalamus is known as the most important neuroendocrine and GPCR‐enriched centre of metabolic regulation in the central nervous system. Here, we performed scRNA‐sequencing of the hypothalamus of 6 weeks old wild‐type mice. A total of 13,977 high‐quality cells were obtained and grouped into 16 cellular clusters (Figure 2A). The classic neuronal markers were employed for cell type annotation of the mouse hypothalamus as shown in Figure 2B. In total, we identified 1,208 neurons (including 626 *Mrap2* expressing neurons). In addition to the classic markers to annotate the cell identities, we also found that some GPCRs (*Grm7, Ntsr2, Calcr1, Gpr17, Lpar1, Gpr84, P2ry6, Ccr1, Adora2a* and *Npy1r*) could be utilized as cell markers for labelling some hypothalamic cell types (Figure 2C).

Next, we examined the co‐expression pattern of all emerging GPCRs with *Mrap2* in mouse hypothalamus. We observed that some GPCRs were highly enriched in *Mrap2*‐positive neurons and oligodendrocytes, moderately expressed in some progenitors, ependymal and endothelial cells (Figures 2D and F). Particularly, a strong positive expression correlation of *Mrap2* and 39 GPCRs (correlation coefficient > .1) was identified in the *Mrap2* neurons of the mouse hypothalamus (Figure 2E), indicating that these co‐expressed GPCRs and *Mrap2* might form functional complex within the same neuronal cell.

To confirm this co‐expression pattern of *Mrap2* and GPCRs in the hypothalamic neurons, we further analysed several published single‐cell datasets of both human and mouse hypothalamus (Figure S2). Consistent with our own dataset, *Mrap2* and many GPCRs clearly co‐expressed in the mouse hypothalamic neurons from the integrative analysis of multiple datasets (Figures S2F and G). Many of these GPCRs in scRNA‐seq data were also included in the selected GPCRs screened in bulk RNA‐seq (Figures 1C, D and 2F). However, a few GPCRs seen in the bulk RNA‐seq datasets failed to be detected in the scRNA‐seq data due to the relatively low sequencing depth. Taken together, we finally summarised the GPCR gene set detected in bulk RNA‐seq (Tables 1 and 2).

**TABLE 1:** Summary of pharmacological regulation of selected GPCR targets by MRAP1

					Constitutive activity		
NO	Gene name	Full name	G protein subclass	Surface translocation	(Gi/Gs)	(Gq)	Maximal agonist response
1	ADRA1B	Adrenergic receptor, alpha 1b	Gq	NS	NT	↓	NT
2	ADRB1	Adrenergic receptor, beta 1	Gs	NS	↓	NT	NT
3	ADRB3	Adrenergic receptor, beta 3	Gs	↑	↑	NT	↓
4	CALCR	Calcitonin receptor	Gs	↓	↓	NT	NT
5	CRHR1	Corticotropin releasing hormone receptor 1	Gs	NS	↑	NT	↓
6	GHSR	Growth hormone secretagogue receptor	Gq	NS	NT	↓	NT
7	GIPR	Gastric inhibitory polypeptide receptor	Gs	NS	NT	↓	NT
8	GPR146	G protein‐coupled receptor 146	unknown	↓	NS	NS	NT
9	GPR151	G protein‐coupled receptor 151	unknown	NS	↑	NT	NT
10	GPR26	G protein‐coupled receptor 26	Gs	NS	↓	NT	NT
11	GPR63	G protein‐coupled receptor 63	Gq	NS	NT	NS	NT
12	GPR75	G protein‐coupled receptor 75	Gq/Gi	NS	↑	↑	NT
13	GPR83	G protein‐coupled receptor 83	Gq	↓	NT	NS	NT
14	GPR88	G protein‐coupled receptor 88	Gi	↑	↓	NT	NT
15	GPRC5B	G protein‐coupled receptor, family C, group 5, member B	Unknown	NS	NS	NS	NT
16	HTR2C	5‐Hydroxytryptamine (serotonin) receptor 2C	Gq	NS	NT	↓	NT
17	LPAR1	Lysophosphatidic acid receptor 1	Gi, Gq	↓	↓	↓	NT
18	MC2R	Melanocortin 2 receptor	Gs	↑	NS	NT	NT
19	MC4R	Melanocortin 4 receptor	Gs	NT	NT	↓	NT
20	NMUR2	Neuromedin U receptor 2	Gi, Gq	NS	↓	NS	NT
21	NPBWR1	Neuropeptides B/W receptor 1	Gi	↑	NS	NT	NT
22	NPFFR1	Neuropeptide FF receptor 1	Gq	↑	NT	NS	NT
23	NPFFR2	Neuropeptide FF receptor 2	Gq	↓	NT	NS	NT
24	NPY1R	Neuropeptide Y receptor Y1	Gi	↑	NS	NT	NT
25	NPY2R	Neuropeptide Y receptor Y2	Gi	↓	↑	NT	NT
26	NTSR2	Neurotensin receptor 2	Gq	NS	NT	NS	NT
27	OPRL1	Opioid receptor‐like 1	Gi	NS	↓	NT	NT
28	OXTR	Oxytocin receptor	Gq	↑	NT	↓	NT
29	PRLHR	Prolactin releasing hormone receptor	Gq	↑	NT	↓	NT
30	PROKR1	Prokineticin receptor 1	Gq	↑	NT	↓	NT
31	PTGER2	Prostaglandin E receptor 2 (subtype EP2)	Gs	↑	↓	NT	↓
32	PTGER3	Prostaglandin E receptor 3 (subtype EP3)	Gi	↑	↑	NT	NT
33	PTGER4	Prostaglandin E receptor 4 (subtype EP4)	Gs	↑	NS	NT	↓
34	SSTR1	Somatostatin receptor 1	Gi	↑	↓	NT	NT
35	SSTR3	Somatostatin receptor 3	Gi	NS	↓	NT	NT
36	SSTR5	Somatostatin receptor 5	Gi	↓	↓	NT	NT
37	TRHR	Thyrotropin releasing hormone receptor	Gq	–	–	–	–
38	C3AR1	Complement component 3a receptor 1	Gi	–	–	–	–
39	CXCR4	Chemokine (C‐X‐C motif) receptor 4	Gi	–	–	–	–
40	GPR150	G protein‐coupled receptor 150	Gs	–	–	–	–
41	GPR176	G protein‐coupled receptor 176	Gs	–	–	–	–
42	GPR19	G protein‐coupled receptor 19	Unknown	–	–	–	–
43	GPR68	G protein‐coupled receptor 68	Gq	–	–	–	–
44	NPY5R	Neuropeptide Y receptor Y5	Gi	–	–	–	–
45	NTSR1	Neurotensin receptor 1	Gq	–	–	–	–
46	PTGER1	Prostaglandin E receptor 1 (subtype EP1)	Gq	–	–	–	–
47	QRFPR	Pyroglutamylated RFamide peptide receptor	Gq	–	–	–	–
48	SSTR2	Somatostatin receptor 2	Gi	–	–	–	–

NT, not tested in this experiment; NS, no significant difference; ↑ up‐regulation; ↓ down‐regulation; – negative results.

**TABLE 2:** Summary of pharmacological regulation of selected GPCR targets by MRAP2

				Constitutive activity			
NO	Gene name	Full name	Surface translocation	(Gi/Gs)	(Gq)	Maximal agonist response	Feeding behaviour
1	ADRA1B	Adrenergic receptor, alpha 1b	NS	NT	↓	NT	–
2	ADRB1	Adrenergic receptor, beta 1	↓	↓	NT	NT	–
3	ADRB3	Adrenergic receptor, beta 3	↑	↓	NT	↓	Orectic
4	C3AR1	Complement component 3a receptor 1	↓	↑	NT	NT	–
5	CALCR	Calcitonin receptor	↓	↓	NT	NT	–
6	CRHR1	Corticotropin releasing hormone receptor 1	↓	↓	NT	↓	–
7	CXCR4	Chemokine (C‐X‐C motif) receptor 4	↓	↑	NT	NT	–
8	FFAR1	Free fatty acid receptor 1	NS	NT	NT	NT	–
9	GHSR	Growth hormone secretagogue receptor	NS	NT	↑	NT	Orectic
10	GIPR	Gastric inhibitory polypeptide receptor	NS	↓	NT	NT	–
11	GLP1R	Glucagon‐like peptide 1 receptor	↓	↓	NT	↓	Anorectic
12	GPR146	G protein‐coupled receptor 146	↓	↑	NS	NT	–
13	GPR150	G protein‐coupled receptor 150	↓	↑	NT	NT	–
14	GPR151	G protein‐coupled receptor 151	↓	↑	NT	NT	–
15	GPR176	G protein‐coupled receptor 176	NS	↑	NT	NT	–
16	GPR19	G protein‐coupled receptor 19	NS	↑	↓	NT	–
17	GPR26	G protein‐coupled receptor 26	NS	↓	NT	NT	–
18	GPR63	G protein‐coupled receptor 63	NS	NT	↓	NT	–
19	GPR75	G protein‐coupled receptor 75	↓	↑	NS	NT	–
20	GPR83	G protein‐coupled receptor 83	↓	NT	NS	NT	–
21	GPR88	G protein‐coupled receptor 88	↑	↑	NT	NT	–
22	GPRC5B	G protein‐coupled receptor, family C, group 5, member B	↓	NS	NS	NT	–
23	HTR2C	5‐Hydroxytryptamine (serotonin) receptor 2C	NS	NT	↓	NT	Anorectic
24	LPAR1	lysophosphatidic acid receptor 1	↓	↑	↓	NT	–
25	MC2R	Melanocortin 2 receptor	Gs				
26	MC4R	Melanocortin 4 receptor	↑	↓	NT	↑	
27	NMUR2	Neuromedin U receptor 2	↓	↑	NS	↑	Anorectic
28	NPBWR1	Neuropeptides B/W receptor 1	↑	↑	NT	NT	–
29	NPFFR1	Neuropeptide FF receptor 1	NS	NT	↓	NT	–
30	NPFFR2	Neuropeptide FF receptor 2	↓	NT	NS	NT	–
31	NPY1R	Neuropeptide Y receptor Y1	↑	NS	NT	NT	Anorectic
32	NPY2R	Neuropeptide Y receptor Y2	↓	↓	NT	NT	Anorectic
33	NPY5R	Neuropeptide Y receptor Y5	↓	↓	NT	NT	Anorectic
34	NTSR2	Neurotensin receptor 2	NS	NT	**→**	NT	–
35	OPRL1	Opioid receptor‐like 1	↓	↓	NT	NT	–
36	PRLHR	Prolactin releasing hormone receptor	↑	NT	↓	NT	–
37	PROKR1	Prokineticin receptor 1	↓	NT	↓	NT	–
38	PTGER1	Prostaglandin E receptor 1 (subtype EP1)	↓	NT	↓	NT	–
39	PTGER2	Prostaglandin E receptor 2 (subtype EP2)	NS	↓	NT	↓	–
40	PTGER3	Prostaglandin E receptor 3 (subtype EP3)	↓	↑	NT	↓	–
41	PTGER4	Prostaglandin E receptor 4 (subtype EP4)	↓	NS	NT	↓	–
42	QRFPR	Pyroglutamylated RFamide peptide receptor	↓	NT	↓	NT	–
43	SSTR2	Somatostatin receptor 2	NS	↓	NT	NT	–
44	SSTR3	Somatostatin receptor 3	↑	↓	NT	NT	–
45	SSTR5	Somatostatin receptor 5	↓	↓	NT	NT	–
46	TRHR	Thyrotropin releasing hormone receptor	↓	NT	↓	NT	–
47	GPR68	G protein‐coupled receptor 68	–	–	–	–	–
48	NTSR1	Neurotensin receptor 1	–	–	–	–	–
49	OXTR	Oxytocin receptor	–	–	–	–	Anorectic
50	SSTR1	Somatostatin receptor 1	–	–	–	–	–

NT, not tested in this experiment; NS, no significant difference; ↑ up‐regulation; ↓ down‐regulation; – negative results.

### 3.3 Co‐expression of GPCRs and MRAP1 in different cell types of both human and mouse adrenal glands

**FIGURE 3:**
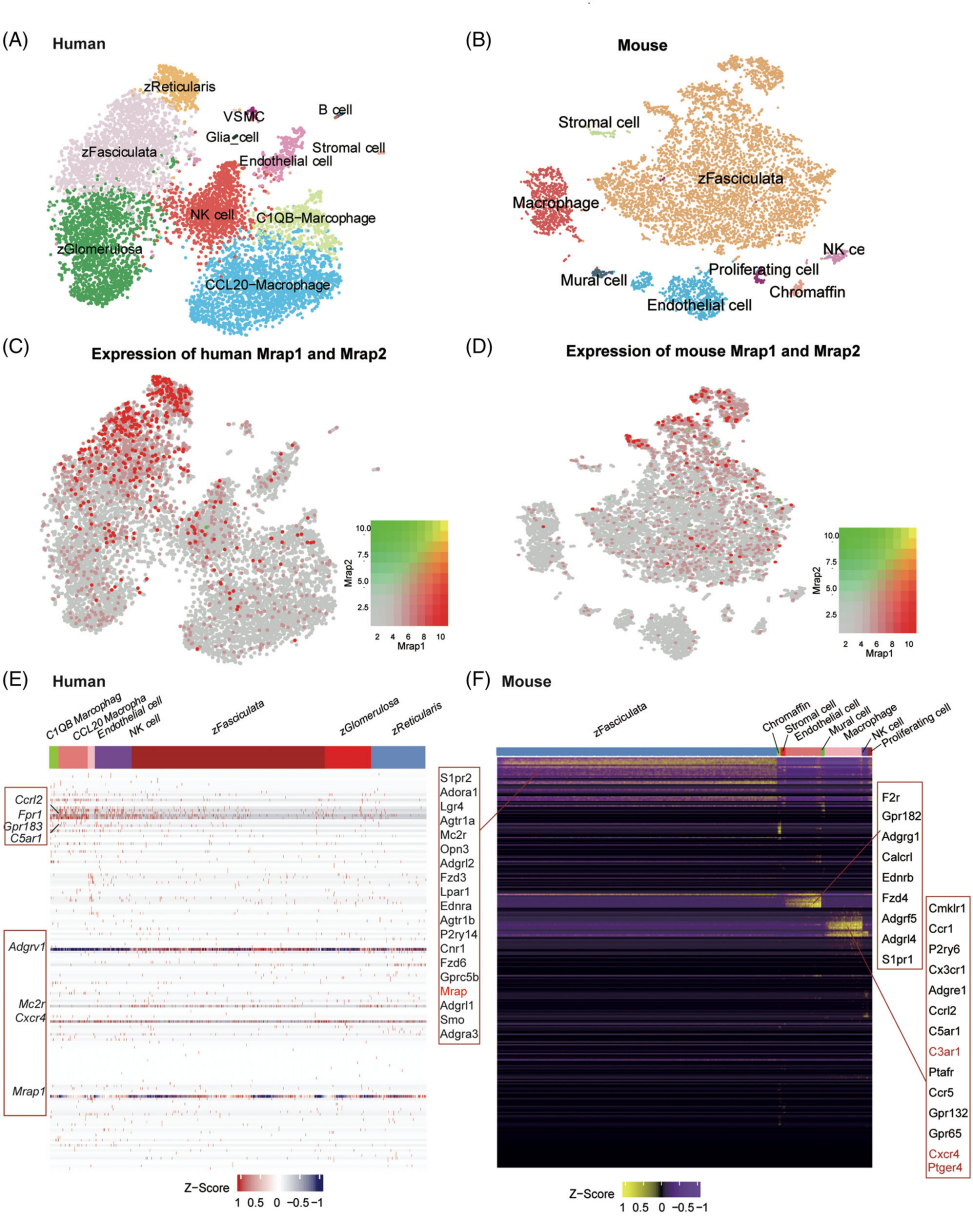
Co‐expression of GPCRs and MRAP2 in different cell types of the human and mouse adrenal gland. (A) tSNE plot of 11 cell types identified in the integrative scRNA‐seq datasets of the human adrenal gland. (B) tSNE plot of eight cell types identified in 6‐week WT mouse adrenal gland generated in this study. (C and D) Expression patterns of MRAPs in human (C) and mouse (D) adrenal gland. MRAP1 and MRAP2 are marked in red and green, respectively. (E and F) Expression heatmap of the detected GPCRs in different cell types of the human (E) and mouse (F) adrenal gland. The highly expressed GPCRs are highlighted in the red box.

**FIGURE 4:**
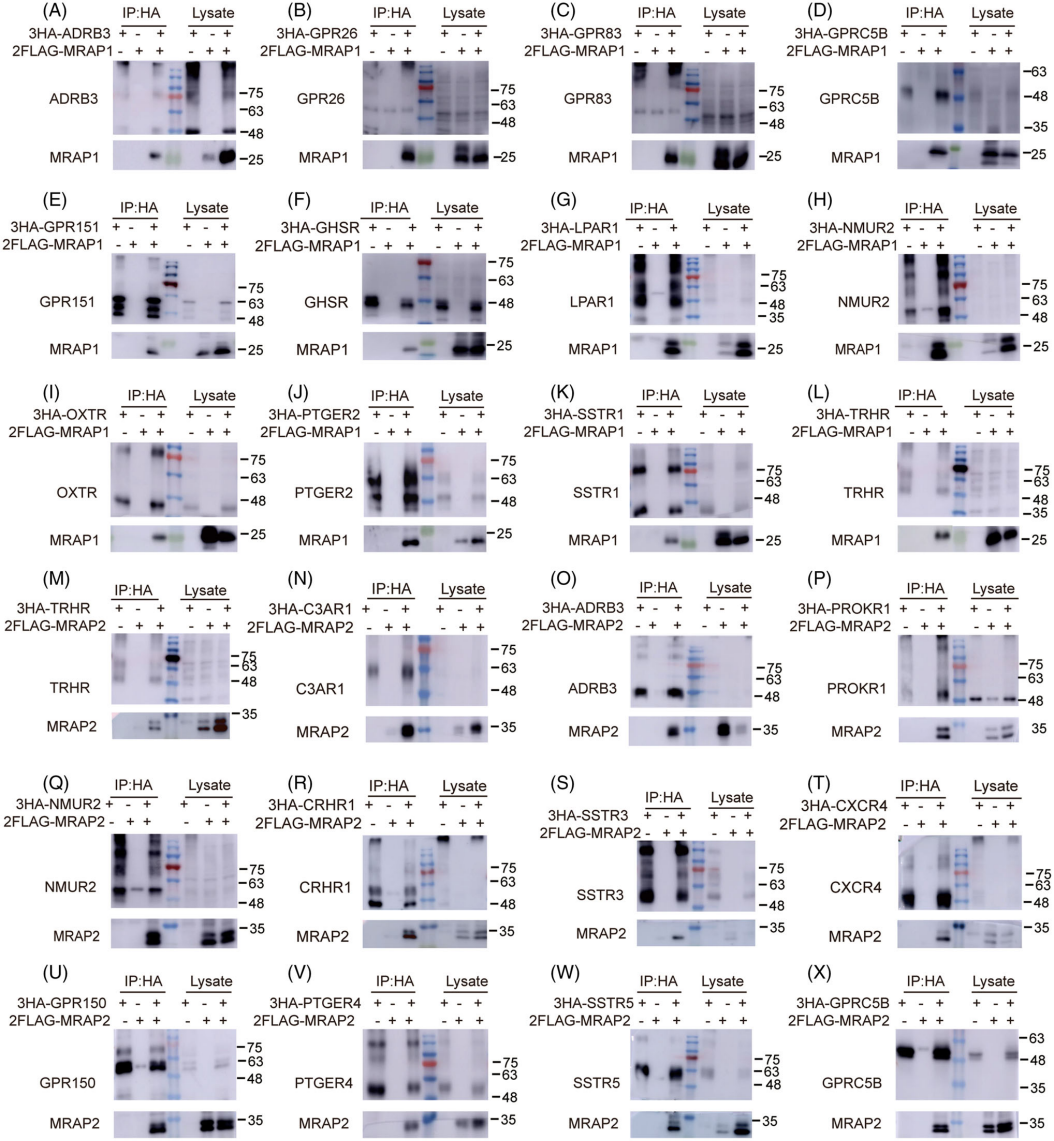
Interaction of GPCRs with MRAP1 or MRAP2. Co‐IP of selected GPCRs with 2Flag‐MRAP1 (A–L) or MRAP2 (M–X) from transfected HEK293T cells. GPCRs were immunoprecipitated using rabbit anti‐HA antibody (IP part on the left) and detected using mouse anti‐HA antibody (upper membrane). MRAP1 (A–L) or MRAP2 (M–X) was detected using mouse anti‐Flag antibody (lower membrane). For a Western blot control, one tenth of the lysate was used. No antibody was used as a control for the IP (lysate part on the right). Co‐IP results of other GPCRs are shown in Figures S5 and S6. Each IP group repeats three times (*n* = 3).

As MRAP1 is highly expressed in the adrenal gland, we next investigated the expression profiles of MRAP1 and GPCRs in both human and mouse adrenal glands at single‐cell resolution. For human adrenal gland, we conducted the integration analysis of the publicly available scRNA‐seq datasets of human adrenal gland (see details in *Material and Methods*). We identified a total of 11 cell types in human adrenal gland scRNA‐seq data (Figure 3A). *MRAP1* was predominantly expressed in two cell types of the human adrenal gland, zFasciculata and zReticularis, while *MRAP2* expression was low in all cell types of the human adrenal gland (Figure 3C). Human *MRAP1* shares its high expression pattern with some GPCRs in several cell types (Figure 4E).

To investigate the expression profiles of MRAP1 and GPCRs in mouse adrenal gland at single‐cell level, we performed scRNA‐seq of the adrenal glands of 6 weeks old wild‐type mice. A total of 9801 high‐quality cells were obtained and grouped into 8 cellular clusters (Figure 3B). We found that *MRAP1* was widely expressed across different cell types, with the highest expression in the mouse zFasciculata cell population (Figure 3D). zFasciculata cell population is mainly associated with glucocorticoid secretion, which is consistent with previous findings that MRAP1 proteins mainly assist the MC2R‐associated glucocorticoid synthesis.^29^ We found that some GPCRs in mouse adrenal gland were also clustered in different adrenal cell types (Figure 4F). Particularly, we identified some highly expressed GPCRs in zFasciculata cells of the mouse adrenal gland, including Mrap1, S1pr2, Adora1, Lgr4, Agtr1a, Mc2r, Opn3, Adgrl2, Fzd3, Lpar1, Ednra, Agtr1b, P2ry14, Cnr1, Fzd6, Gprc5b, Adgrl1, Smo and Adgra3. We also performed the integration analysis of public available scRNA‐seq datasets of mouse adrenal gland and identified a similar expression pattern of *MRAP1* and *GPCRs* in the corresponding cell types of the mouse adrenal glands (Figure S3).

Interestingly, we found that the overall GPCR expression levels and numbers of detected GPCRs in adrenal gland are much lower than that in hypothalamus. In addition, the co‐expression pattern of GPCRs with MRAP2 in hypothalamus is much clearer than that with MRAP1 in adrenal gland. For instance, we found that only a few of the GPCRs (like C3ar1, Cxcr4 and Ptger4) were highly expressed in the adrenal scRNA‐seq data are in our 48 GPCR gene sets (Figure 3F), which also indicates that the interaction network between MRAP2 in hypothalamus is stronger than that between MRAP1 in adrenal gland with GPCRs. Therefore, the hypothalamus should be a more critical tissue for GPCRs to express and function by interacting with MRAPs, especially MRAP2. Overall, these data suggested that *MRAP1* and these co‐expressing GPCRs may form functional complexes within the same endocrine cells to regulate glucocorticoid secretion in human and mouse adrenal glands.

### 3.4 Interactions of MRAP proteins with sorted GPCRs

Next, we performed co‐immunoprecipitation (co‐IP) assays to examine the ability of sorted GPCRs to form a protein complex with MRAP1 and MRAP2 proteins. GPCRs were labelled with 3xHA tag and MRAP1 or MRAP2 was labelled with 2xFlag tag. Indicated plasmids were transfected into HEK293T cells and the total plasmid amounts were kept consistent in each experiment by adding empty vectors. Next, total proteins were pulled down with a monoclonal anti‐HA antibody. The precipitated proteins from the cell lysates were sorted by Western blot with anti‐HA antibody for GPCRs or anti‐Flag antibody for MRAP1 or MRAP2. As shown in Figures 4 and S4 and S5, we found that a fraction of MRAP1 and MRAP2 co‐precipitated with most sorted GPCRs. Thirty‐six and 46 GPCRs have direct interactions with MRAP1 and MRAP2, respectively. Consistent with the previous study, MC2R could interact with both MRAP1 and MRAP2 in vitro.^30^ Several GPCRs, MC4R, PKR1 and GHSR1a, have been reported in previous studies to be regulated by MRAP2^12^, ^15^, ^31^ and we have verified the interactions between these receptors and MRAP2. Notably, TRHR, C3AR1, CXCR4, FFAR1, GPR150, GPR176, GPR19, NPY5R, PTGER1, QRFPR and SSTR2 interacted with MRAP2 only. Conversely, we only observed the protein complex between OXTR or SSTR1 and MRAP1, not MRAP2. GPR68 and NTSR1 did not interact with MRAP1 or MRAP2. These results suggested that MRAP2 could interact with a wider GPCR network than MRAP1 and strongly supported the co‐expression correlation ranking from bioinformatic analysis in Figure 2.

**FIGURE 5:**
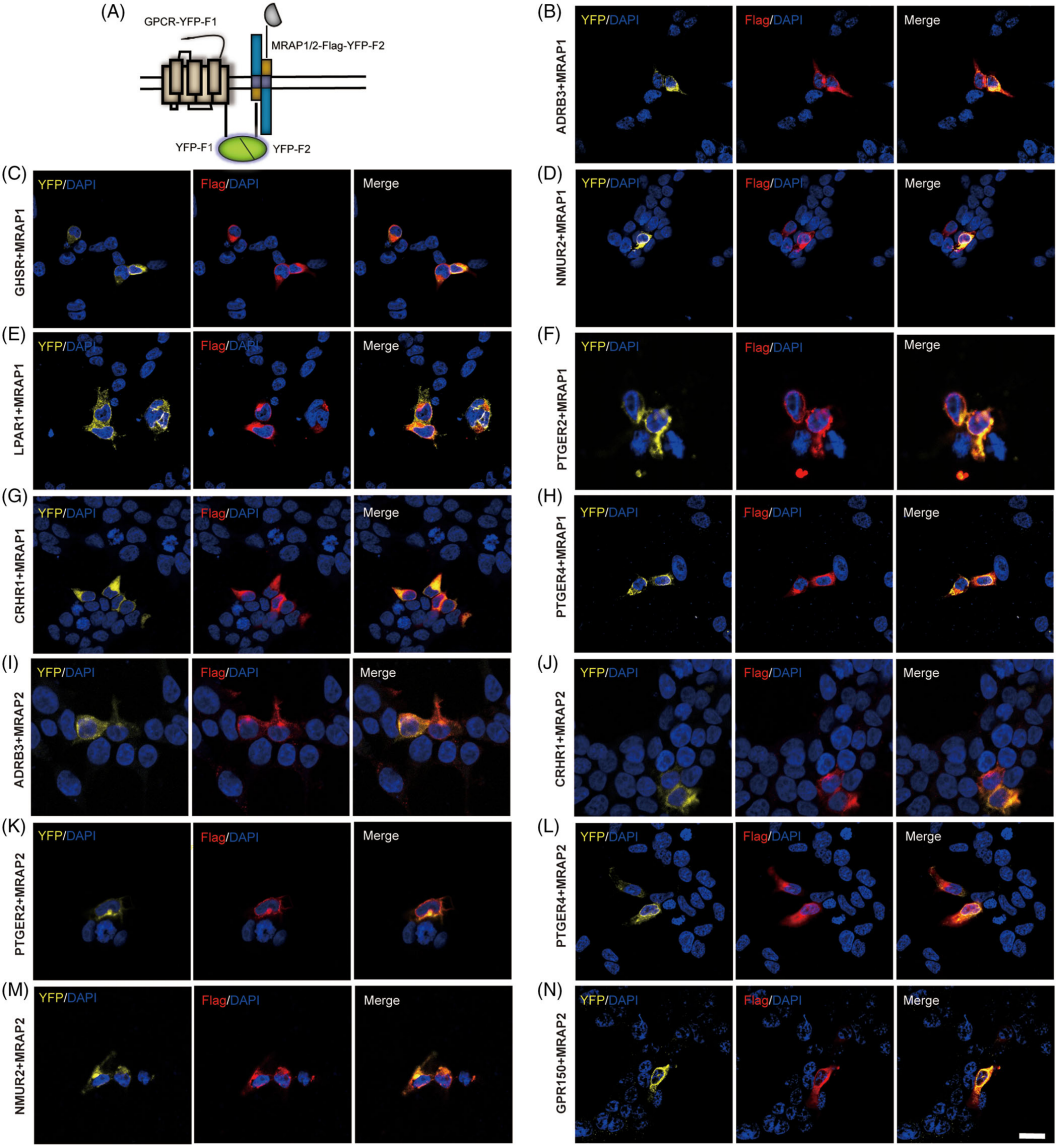
Co‐localisation of selected GPCRs and MRAP proteins in cells. (A) Schematic representation of bimolecular fluorescence complementation (BiFC) between YFP fragments fused to GPCRs and MRAP proteins. HEK293T cells were transfected with MRAP1‐Flag‐F2 (B–H) or MRAP2‐Flag‐F2 (I–N). Nuclei stained with DAPI are shown in blue and YFP fluorescence in yellow. MRAP1 and MRAP2 emitted red fluorescence after incubation with Flag antibody and Alexa Fluor594 secondary antibody. Each YFP group was repeated at least twice, with three fields of view selected.

To further determine the interactions in live cells and the subcellular localisation of these complexes, we performed bimolecular fluorescence complementation assays with selected GPCRs and MRAPs, as described previously.^32^ Basically, the C‐terminus of these GPCRs were fused to a fragment of YFP which called F1, while the C‐end of MRAP1 or MRAP2 were fused to the complementary fragment of YFP (F2). With this approach, YFP fluorescence can only be detected if the fused GPCR and MRAP1 or MRAP2 are co‐expressed and come close to allow the YFP to complement (Figures 5 and S6). Thus, these results suggested that selected GPCRs interacted with either of MRAP1 and MRAP2 or both in live cells, in accordance with the results of co‐IP experiments.

### 3.5 Pharmacological modulation of MRAP proteins on GPCR signalling

**FIGURE 6:**
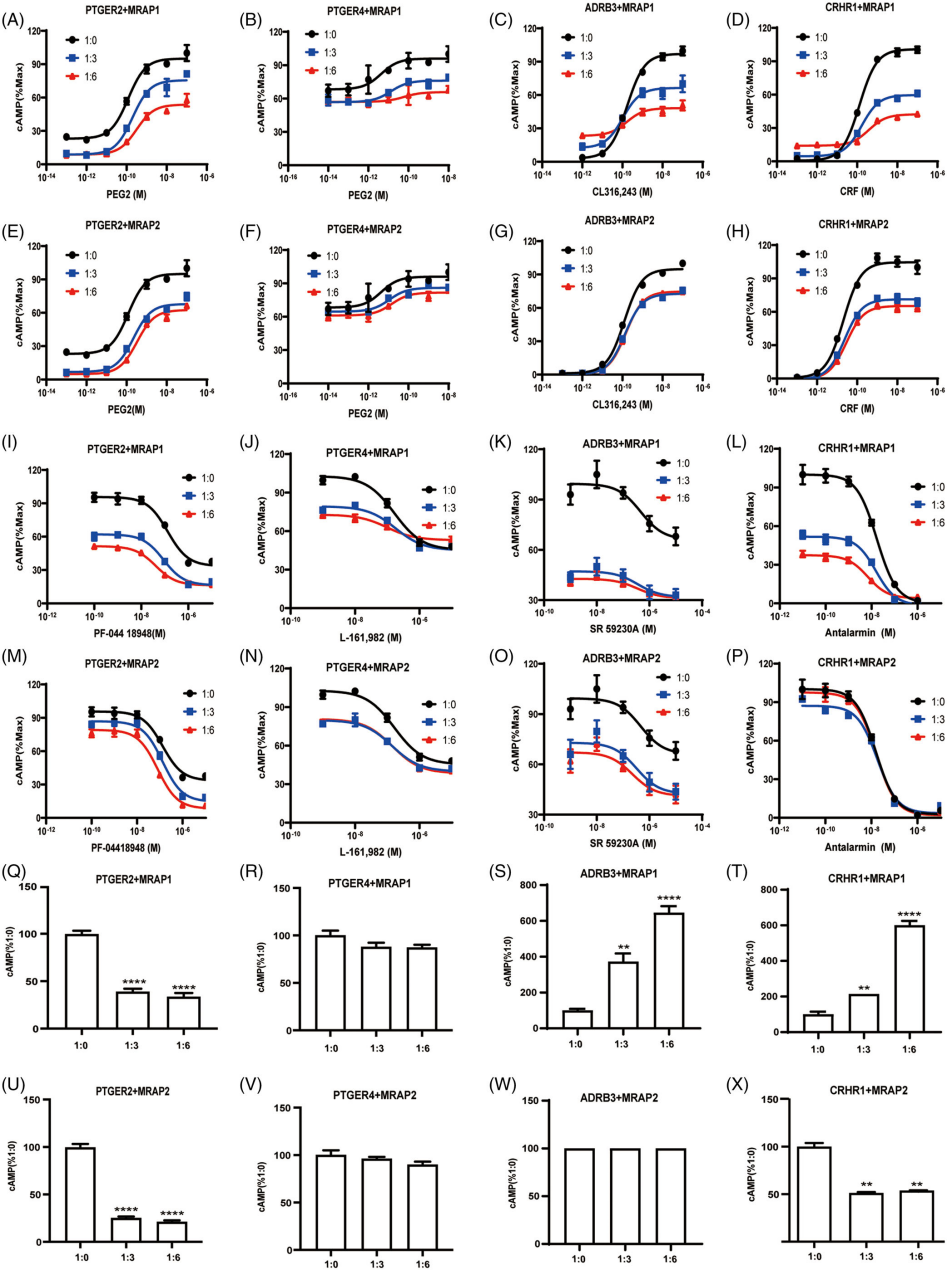
Pharmacological modulation of selected GPCRs signalling by MRAP proteins. (A‐H) Modulation of agonist‐simulated signalling by MRAP proteins. Concentration–response curves of agonist‐induced cAMP production of PTGER2, PTGER4, ADRB3 and CRHR1 in HEK293T cells upon transfection with MRAP1 (A–D) or MRAP2 (E–H) in ratio of 1:0, 1:3 and 1:6. Each dot represents the mean ± SEM of three replicates (*n* = 3). (I–P) Modulation of antagonist competition binding by MRAP proteins. Concentration–response curves of antagonist‐induced cAMP production of PTGER2, PTGER4, ADRB3 and CRHR1 in HEK293T cells upon transfection with MRAP1 (I–L) or MRAP2 (M–P) in ratio of 1:0, 1:3 and 1:6. Each dot represents the mean ± SEM of three replicates (*n* = 3). (Q–X) Modulation of the constitutive activities of selected GPCRs by MRAP proteins. Measurement of the constitutive activities of PTGER2, PTGER4, ADRB3 and CRHR1 in the presence of various doses of MRAP1 (Q–T) or MRAP2 (U–X) proteins. Modulation of the constitutive activities results of other GPCRs are shown in Figure S8. One‐way ANOVA with post hoc Tukey's test. *p < .05, **p < .01, ***p < .001, ****p < .0001. Each data column represents the mean ± SEM of three replicates (*n* = 3).

Luciferase reporter assays were then performed to further verify the roles of MRAP1 and MRAP2 on the pharmacological properties of these GPCRs. In this study, we selected several GPCRs that were strongly related to energy metabolism (Table S1) and tested the effects of MRAP1 and MRAP2 on their activity challenged with various concentrations of agonists. HEK293T cells were transfected with several plasmids, including GPCR, either MRAP1 or MRAP2 and a luciferase (luc) encoding plasmid under the control of cAMP‐responsive elements (CREs) promoter to detect Gs signalling of ADRB3, CRHR1, PTGER2 and PTGER4 (Figure 6). The total amount of protein used in the experiment was identical. This method of driving luc expression by cAMP yields results consistent with direct measurement of cAMP level.

Based on the information in the UNIPROT database, we classified all screened GPCRs into three categories: Gs/Gi, Gq and unknown. Then, we determined their constitutive activity in the presence of MRAP1 or MRAP2 according to the classification. Among them, we measured the constitutive activity of Gs/Gi and Gq pathway both for the unknown GPCRs. The calcium signalling was assessed by NFAT luciferase activity. We found that both constitutive activity and PEG2‐inducible activity of PTGER2 and PTGER4 were suppressed by increasing expression of MRAP1 or MRAP2 (Figures 6A, B, E and F), which suggested that MRAP1 and MRAP2 stabilised an inactive conformation of PTGER2 and PTGER4. However, the substantial cAMP response of ADRB3 and CRHR1 did not change, but their maximum activity was reduced with overexpressed MRAP2 (Figures 6C, D, G and H). Furthermore, substantial cAMP response of ADRB3 and CRHR1 decreased, but the maximum activity reduced when overexpressed MRAP1 (Figures 6C, G, S and W). These results indicated that MRAP1 or MRAP2 inhibited the signal transduction of these GPCRs. Moreover, as seen in Tables 3 and 4, the EC50 did not change too much in the presence of MRAP1 or MRAP2, but it did have a dose effect on the signal transduction of these GPCRs. The activation of MC2R by ACTH requires the presence of MRAP1. MC2R and MC4R are significantly altered in EC50 under the regulation of MRAP1 or MRAP2,^7^, ^33^, ^34^ but there are similar studies showing that MRAP2 inhibits overall cAMP levels of some GPCRs like PKR1^12^ and has little effect on their EC50. To evaluate whether their antagonist could block agonist simulated activation of these GPCRs, we also supplemented different concentrations of antagonists in the presence of EC80 agonist. As shown in Figures 6I–P, MRAP1 and MRAP2 both inhibited the activity of ADRB3, CRHR1 PTGER2 and PTGER4. Overall, we found that the addition of MRAP1 and MRAP2 either affect the EC50 or the maximal response of these GPCRs to their ligands, implying that MRAP proteins may resemble allosteric modulators of GPCRs and thus could exert the regulatory role in a composite manner (Tables 3 and 4).

**TABLE 3:** Statistical analysis of agonist stimulated GPCRs activity in the presence of different doses of MRAP1 or MRAP2

		LogIC50			*p* Value for *V*_max_ comparison		
Data statistics of Figures 7I–P		1:0	1:3	1:6	1:0 vs. 1:3	1:0 vs. 1:6	1:3 vs. 1:6
Figure 7I	ADRB3:MRAP1	−6.332 ± 0.59	−6.444 ± 0.75	−6.458 ± 0.62	<.0001	<.0001	.1454
Figure 7J	CRHR1:MRAP1	−8.011 ± 0.13	−7.725 ± 0.17	−7.428 ± 0.21	<.0001	<.0001	.1861
Figure 7K	PTGER2:MRAP1	−6.879 ± 0.16	−7.055 ± 0.16	−7.391 ± 0.11	<.0001	<.0001	<.0001
Figure 7J	PTGER4:MRAP1	−6.782 ± 0.16	−6.742 ± 0.22	−7.008 ± 0.35	.0007	.0144	.8359
Figure 7M	ADRB3:MRAP2	−6.332 ± 0.59	−6.492 ± 0.70	−6.646 ± 0.63	<.0001	<.0001	.0001
Figure 7N	CRHR1:MRAP2	−7.985 ± 0.14	−7.789 ± 0.21	−7.568 ± 0.17	.0054	<.0001	<.0001
Figure 7O	PTGER2:MRAP2	−6.879 ± 0.16	−6.881 ± 0.12	−7.093 ± 0.13	<.0001	<.0001	<.0001
Figure 7P	PTGER4:MRAP2	−6.782 ± 0.16	−6.854 ± 0.21	−6.842 ± 0.18	<.0001	<.0001	.9769

Values were expressed as the mean ± SEM of at least three independent experiments. Two‐way ANOVA with Tukey post‐test was applied in the statistical analysis.

**TABLE 4:** Statistical analysis of antagonist inhibited GPCR activity in the presence of different doses of MRAP1 or MRAP2

NO	Gene name	Full name	Interaction with MRAP1	Interaction with MRAP2	Animal studies	Human studies
1	ADRA1B	Adrenergic receptor, alpha 1b	Yes	Yes	Overexpression of *Adra1b* in mice: dilated cardiomyopathy, premature death (Lemire et al., 2001)	GWAS, genetic link between ADRA1B and Rheumatoid arthritis (Plenge et al., 2007)
2	ADRB1	Adrenergic receptor, beta 1	Yes	Yes	*Adrb1*−/− mice: increased body weight, impaired adaptive thermogenesis, increased circulating leptin level, decreased oxygen consumption (Bachman et al., 2002)	GWAS, genetic link between ADRB1 and blood pressure, attention deficit hyperactivity disorder (Lasky‐Su et al., 2008; Wain et al., 2011)
3	ADRB3	Adrenergic receptor, beta 3	Yes	Yes	*Adrb3*−/− mice: increased total body fat amount, abnormal physiological response to xenobiotic (Susulic et al., 1995)	None
4	C3AR1	Complement component 3a receptor 1	No	Yes	*C3ar1*−/− mice: decreased circulating free fatty acids level, decreased circulating phospholipid level (Yu et al., 2012). TLQP‐21 treatment decreases body weight and fat mass in HFD mice by C3ar1 activation (Cero et al., 2017)	None
5	CALCR	Calcitonin receptor	Yes	Yes	*Calcr*−/− mice: abnormal circulating calcium level, abnormal circulating alkaline phosphatase level (https://www.mousephenotype.org/)	GWAS, genetic link between CALCR and type II diabetes mellitus and related traits (Florez et al., 2007)
6	CRHR1	Corticotropin releasing hormone receptor 1	Yes	Yes	*Crhr1*−/− mice: adrenal gland hypoplasia, abnormal hormone level, impaired HPA axis response to physical‐restraint stress (Smith et al., 1998)	GWAS, genetic link between CRHR1 and Alzheimer disease, bone density, body height and blood glucose (Harold et al., 2009; Lango Allen et al., 2010; Rivadeneira et al., 2009; Saxena et al., 2010)
7	CXCR4	Chemokine (C‐X‐C motif) receptor 4	No	Yes	*Cxcr4*−/− mice: increased subcutaneous adipose tissue amount, increased susceptibility to diet‐induced obesity, impaired adaptive thermogenesis, decreased oxygen consumption, increased susceptibility to diet‐induced obesity (Yao et al., 2014)	None
8	GHSR	Growth hormone secretagogue receptor	Yes	Yes	*Ghsr*−/− mice: decreased body weight, decreased circulating insulin‐like growth factor I level, abnormal growth hormone level, abnormal food intake (Sun et al., 2004)	GWAS, genetic link between GHSR and body height(Lango Allen et al., 2010)
9	GIPR	Gastric inhibitory polypeptide receptor	Yes	Yes	*Gipr*−/− mice: abnormal pancreas secretion, increased circulating glucose level, decreased circulating insulin level, impaired glucose tolerance (Preitner et al., 2004)	GWAS, genetic link between GIPR and body height (Saxena et al., 2010; Speliotes et al., 2010)
10	GPR146	G protein‐coupled receptor 146	Yes	Yes	*Gpr146*−/− mice: decreased circulating cholesterol level, decreased circulating HDL cholesterol level, decreased circulating triglyceride level, decreased circulating iron level, increased circulating alanine transaminase level (https://www.mousephenotype.org/)	None
11	GPR150	G protein‐coupled receptor 150	No	Yes	*Gpr150*−/− mice: shortened PR interval level (https://www.mousephenotype.org/)	None
12	GPR151	G protein‐coupled receptor 151	Yes	Yes	No abnormal phenotype detected (https://www.mousephenotype.org/)	None
13	GPR176	G protein‐coupled receptor 176	No	Yes	*Gpr176*−/− mice: abnormal head morphology, increased circulating creatinine level, increased blood urea nitrogen level (https://www.mousephenotype.org/)	GWAS, genetic link between GPR176 and Amyotrophic Lateral Sclerosis (Schymick et al., 2007)
14	GPR19	G protein‐coupled receptor 19	No	Yes	*Gpr19*−/− mice: increased anxiety‐related response (https://www.mousephenotype.org/)	None
15	GPR26	G protein‐coupled receptor 26	Yes	Yes	*Gpr26*−/− mice: increased susceptibility to diet‐induced obesity, increased total body fat amount, increased food intake, decreased circulating ghrelin level, increased circulating insulin level, increased circulating leptin level, increased circulating cholesterol level, increased circulating triglyceride level, decreased energy expenditure, abnormal glucose homeostasis, increased circulating glucose level, impaired glucose tolerance (Chen et al., 2012)	None
16	GPR63	G protein‐coupled receptor 63	Yes	Yes	*Gpr63*−/− mice: increased anxiety‐related response (https://www.mousephenotype.org/)	None
17	GPR75	G protein‐coupled receptor 75	Yes	Yes	*Gpr75*−/− mice: thin with improved glucose homeostasis, resistance to weight gain and improved glycemic control in a high‐fat diet model (Powell et al., 2022)	Protein‐truncating variants in GPR75 individuals have lower BMI and lower odds of obesity in the heterozygous state
18	GPR83	G protein‐coupled receptor 83	Yes	Yes	*Gpr83*−/− mice: protected from obesity and glucose intolerance when challenged with a high‐fat diet (Müller et al., 2013)	None
19	GPR88	G protein‐coupled receptor 88	Yes	Yes	*Gpr88*−/− mice: reduced adiposity, suppressed food intake, lower energy expenditure, unchanged body weight. Deregulation in glucose tolerance and insulin responsiveness(Lau et al., 2017)	GWAS, genetic link between GPR88 and BPD (Del Zompo et al., 2014)
20	GPRC5B	G protein‐coupled receptor, family C, group 5, member B	Yes	Yes	*Gprc5b*−/− mice: altered spontaneous activity pattern, decreased response to a new environment (Sano et al., 2011)	GWAS, genetic link between GPRC5B and body mass index (Speliotes et al., 2010)
21	HTR2C	5‐Hydroxytryptamine (serotonin) receptor 2C	Yes	Yes	*Htr2c*−/− mice: decreased body weight, decreased food intake (Morabito et al., 2010)	None
22	LPAR1	Lysophosphatidic acid receptor 1	Yes	Yes	*Lpar1*−/− mice: decreased body fat mass, decreased body weight, decreased bone mineral density (Potter et al., 2016)	GWAS, genetic link between LPAR1 and brain glutamate concentrations (Baranzini et al., 2010)
23	MC2R	Melanocortin 2 receptor	Yes	Yes		
24	MC4R	Melanocortin 4 receptor	Yes	Yes	*Mc4r*−/− mice: obese (Shah et al., 2014)	GWAS, genetic link between MC4R and body mass index (Speliotes et al., 2010)
25	NMUR2	Neuromedin U receptor 2	Yes	Yes	*Nmur2*−/− mice: decreased body weight, decreased food intake, decreased susceptibility to diet‐induced obesity (Peier et al., 2009)	None
26	NPBWR1	Neuropeptides B/W receptor 1	Yes	Yes	*Npbwr1*−/− mice: increased circulating insulin and leptin level, abnormal lipid homeostasis, increased susceptibility to diet‐induced and age‐related obesity (Ishii et al., 2003)	None
27	NPFFR1	Neuropeptide FF receptor 1	Yes	Yes	*Npffr1*−/− mice: abnormal pituitary secretion, increased susceptibility to weight loss, decreased luteinising hormone level (León et al., 2014)	None
28	NPFFR2	Neuropeptide FF receptor 2	Yes	Yes	*Npffr2*−/− mice: abnormal brown adipose tissue thermogenesis, decreased body weight, decreased food intake (Zhang et al., 2018)	None
29	NPY1R	Neuropeptide Y receptor Y1	Yes	Yes	*Npy1r*−/− mice: decreased epididymal fat pad weight, decreased total fat pad weight, slow postnatal weight gain (Bertocchi et al., 2011)	None
30	NPY2R	Neuropeptide Y receptor Y2	Yes	Yes	*Npy2r*−/− mice: decreased body weight, decreased white adipose tissue amount, abnormal eating behaviour (Sainsbury et al., 2003)	None
31	NPY5R	Neuropeptide Y receptor Y5	No	Yes	*Npy5r*−/− mice: decreased food intake, increased susceptibility to diet‐induced obesity, obese (Kanatani et al., 2000)	None
32	NTSR2	Neurotensin receptor 2	Yes	Yes	*Ntsr2*−/− mice: abnormal gait, decreased startle reflex (Feifel et al., 2010) increased thermal nociceptive threshold (Maeno et al., 2004)	None
33	OPRL1	Opioid receptor‐like 1	Yes	Yes	*Oprl1*−/− mice: decreased thermal nociceptive threshold, abnormal passive avoidance behaviour, decreased chemically‐elicited anti‐nociception (Depner et al., 2003; Noda et al., 1998)	GWAS, genetic link between OPRL1 and ulcerative colitis (Anderson et al., 2011)
34	PRLHR	Prolactin releasing hormone receptor	Yes	Yes	*Prlhr*−/− mice: decreased food intake, increased body weight, increased circulating glucose level (Bjursell et al., 2007)	None
35	PROKR1	Prokineticin receptor 1	Yes	Yes	*Prokr1*‐/‐ in mice: paired development of gonadotropin‐releasing hormone neurons and infertility (Zhao et al., 2019)	None
36	PTGER1	Prostaglandin E receptor 1 (subtype EP1)	No	Yes	*Ptger1*‐/‐ mice: manifest behavioural disinhibition, including impulsive aggression with defective social interaction, impaired cliff avoidance, and an exaggerated acoustic startle response (Matsuoka et al., 2005)	None
37	PTGER2	Prostaglandin E receptor 2 (subtype EP2)	Yes	Yes	*Ptger2−*/− mice: *r*esting systolic blood pressure (BP) (Sato et al., 2007) Mutations of PTGER2 in mice: adenoma growth is suppressed (Seno et al., 2002)	None
38	PTGER3	Prostaglandin E receptor 3 (subtype EP3)	Yes	Yes	*Ptger3−/−* mice: increased frequency of feeding during the light cycle of the day and develop an obese phenotype under a normal fat diet fed ad libitum. stimulation of leptin release from adipose tissue to involve actions (Sanchez‐Alavez et al., 2007)	None
39	PTGER4	Prostaglandin E receptor 4 (subtype EP4)	Yes	Yes	*Ptger4−/−* mice: associated with increased fibrosis, reduced EF and dilated cardiomyopathy (Harding et al., 2010).	GWAS, genetic link between PTGER4 and Crohn Disease (Franke et al., 2010; Hindorff et al., 2009)
40	QRFPR	Pyroglutamylated RFamide peptide receptor	No	Yes	*Qrfpr−/−* mice: a thinned osteochondral growth plate, a thickening of trabecular branches, and a reduction in osteoclast number, suggestive of an early arrest of osteochondral bone formation (Baribault et al., 2006) Mutations of *Qpfpr* in mice: exhibit nephropathy indicative of uricase deficiency (Cook et al., 2001)	GWAS, genetic link between QRFPR and narcorlepsy in a Japanese population (Koike et al., 2009; Miyagawa et al., 2008)
41	SSTR2	Somatostatin receptor 2	No	Yes	*Sstr2−/−* mice: the numbers of somatostatin cells were reduced in the antrum (−55%) and increased in the oxyntic mucosa (35%) (Zhao et al., 2008)	GWAS, genetic link between SSTR2 and body mass index and waist circumference in the Farmingham Heart study (Fox et al., 2007; Hindorff et al., 2009)
42	SSTR3	Somatostatin receptor 3	Yes	Yes	Mutations of *Sstr3* in mice: the Koa phenotype in mice (Fantauzzo et al., 2008)	None
43	SSTR5	Somatostatin receptor 5	Yes	Yes	*Sst−/−* mice: showed enhanced insulin and glucagon secretory responses in vivo (Hauge‐Evans et al., 2009)	None
44	TRHR	Thyrotropin releasing hormone receptor	No	Yes	*Trhr1−*/− mice: displayed a higher body weight loss and a delayed reduction in locomotor activity upon fasting (Mayerl et al., 2015)	None
45	GPR68	G protein‐coupled receptor 68	No	No	*GPR68−*/− mice: showed brown adipose tissue (BAT) abnormality (Li et al., 2009)	None
46	NTSR1	Neurotensin receptor 1	No	No	*NTSR1−*/− mice: displayed increased white adipose tissue amount, increased body weight and body weight (Remaury et al., 2002)	GWAS, genetic link between SSTR2 and Breast Neoplasms and Ulcerative colitis
47	OXTR	Oxytocin receptor	Yes	No	*Oxtr−/−* mice: demonstrated defects in lactation and maternal nurturing, emitted fewer ultrasonic vocalisations in response to social isolation, showed deficits in social discrimination and elevated aggressive behaviour, showed similar high levels of aggression (Takayanagi et al., 2005)	None
48	SSTR1	Somatostatin receptor 1	Yes	No	*Sst−/−* mice: showed enhanced insulin and glucagon secretory responses in vivo (Hauge‐Evans et al., 2009)	None

Next, we measured the constitutive Gs(cAMP) or Gq(Ca2+) activities of selected GPCRs in the presence of various doses of MRAP1 or MRAP2 Like MC4R, most of the identified GPCR targets exhibited basal constitutive activity in the absence of agonists. MRAP proteins dramatically altered the basal intracellular cAMP or Ca2+ levels of most GPCRs (Figures 6 and S7). These results suggested that MRAP1 and MRAP2 could modulate the constitutive activity of these GPCRs.

### 3.6 Alteration of trafficking and ERK pathway of selected GPCRs in the presence of MRAP proteins

**FIGURE 7:**
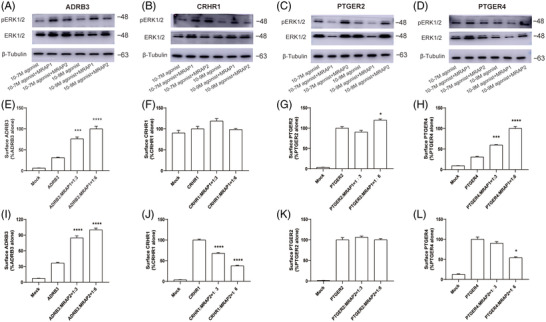
ERK signalling and surface expression of GPCRs in the presence of MRAPs. (A–D) Western blot for ERK1/2 and pERK1/2, and Tubulin was used as reference control. The samples order from left to right were: GPCR only (with 10^‐7^ M agonist), GPCR+MRAP1 (with 10^‐7^ M agonist), GPCR+MRAP2 (with 10^‐7^ M agonist), GPCR only (with 10^‐9^ M agonist), GPCR+MRAP1 (with 10^‐9^ M agonist), GPCR+MRAP2 (with 10^‐9^ M agonist). (E–L) Surface expression of selected GPCRs in HEK293 cells transfected with empty vector, MRAP1 or MRAP2 at 1:3 and 1:6 ratio using cell ELISA assays. One‐way ANOVA with post hoc Turkey test. ns, no significant change; *p < .05, **p < .01, ***p < .001, ****p < .0001. ELISA results of other GPCRs are shown in Figure S9. Each data column represents the mean ± SEM of three replicates (*n* = 3).

Given that the MRAP proteins interacted with most GPCRs, we next investigated the mechanism by which MRAP proteins regulated these GPCRs. MC4R activation, which is primarily regulated by MRAP2, leads to cAMP synthesis and activates the downstream signalling pathway and phosphorylates extracellular signal‐regulated kinase 1 and 2 (ERK1/2).^35^, ^36^, ^37^, ^38^, ^39^ To test the effect of MRAPs on the ERK signalling downstream of the selected GPCRs, we transfected several GPCRs with or without MRAP1 or MRAP2 and examined their expression levels of ERK1/2 and phosphorylated ERK1/2. We found that the addition of MRAP1 or MRAP2 did cause changes in the ERK signalling of selected GPCRs (Figures 7A–D).

Since the presence of MRAP proteins affected the cell surface translocation of MCRs, we then tested whether MRAP proteins could modulate the post‐translational cell surface trafficking of the receptor. Next, we transfected HEK293T cells with 3HA‐tagged GPCRs with either MRAP1, MRAP2 or a blank vector. We then measured the cell‐surface expression of GPCR in non‐permeabilised fixed‐cells with enzyme‐linked immunosorbent assay (ELISA). As shown in Figures 7 and S9, MRAP proteins showed a distinct effect on the cell surface expression of GPCRs. Consistent with the results in previous studies,^34^, ^40^ we found that both MRAP1 and MRAP2 significantly reduced the membrane transport of MC4R. We found that MRAP1 increased the membrane trafficking of most GPCRs, while MRAP2 decreased their surface expression. In addition, the surface expression trends of MRAP1 and MRAP2 on GPR83, NPY2R, SSTR5, NPFFR2, LPAR1, GPR88, NPY1R, NPBWR1 and PRLHR were consistent. Among them, both MRAP1 and MRAP2 reduced the surface level expression of GPR83, NPY2R, SSTR5, NPFFR2 and LPAR1, while both increased GPR88, NPY1R, NPBWR1 and PRLHR. However, the trafficking effect of MRAP1 and MRAP2 on SSTR1, PTGER3, PTGER4 and PROKR1 was the opposite. The surface expression of these GPCRs was elevated in the presence of MRAP1 and reduced in the presence of MRAP2 (Figures 7 and S8). Moreover, we also found that the surface level of several GPCRs was not affected by the MRAP protein, such as GPR26, GPR63, GPCR5B, GHSR and HTR2C (Figure S8). Taken together, MRAP1 and MRAP2 proteins regulated the trafficking of most sorted GPCRs in vitro.

### 3.7 In vivo functional validation of MRAP2–GPCR complexes

**FIGURE 8:**
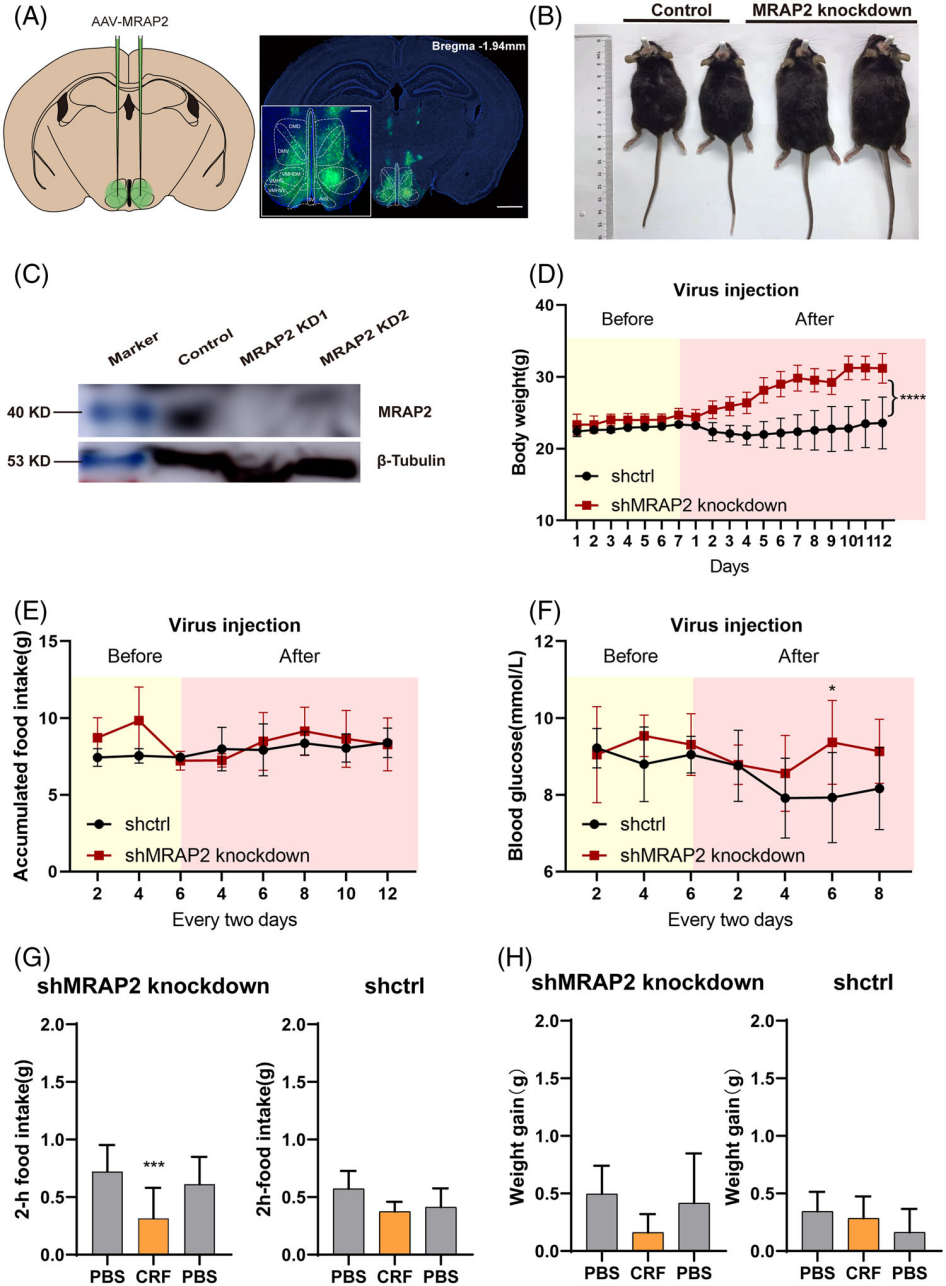
Physiological evaluation of MRAP2–CRHR1 complex function in vivo. (A) Representation of viral administration and expression of shNRAP2 in mouse hypothalamus. Scale bar, 500 μm. (B) Increased body weight gain in shMRAP2 knockdown mice compared to control group. (C) Representation of the suppression of MRAP2 expression by Western blot of shRNA‐control or shRNA‐MRAP2. (D) Body weight change, (E) food intake and (F) blood glucose measurement in MRAP2‐knockout (*n* = 8) and GFP control (*n* = 8) mice. (G) Body weight change and (H) food intake in MRAP2‐knockout (*n* = 4) mice and GFP control mice (*n* = 4) upon CRF or PBS ICV injection.

MRAP2 deletion or loss‐of‐function mutation resulted in obesity syndrome in mice and human.^7^ We next assessed the in vivo physiological phenotype when the endogenous MRAP2 was knocked down. We bilaterally injected pscAAV‐U6‐MRAP2‐CMV‐GFP‐tWPA into the arcuate nucleus of C57BL/6J mice (Figures 8A–C). Weight gain (Figure 8D) of MRAP2 knockdown group significantly increased, but no significant change was seen for accumulative food intake (Figure 8E) or blood glucose level (Figure 8F). In a previous study, Li et al.^41^ found that the number of Mc4r neurons in the hypothalamic paraventricular nucleus exhibited a large amount of isotopic labelling (22.5% ± 2.6) with CRH neurons. Corticotropin‐releasing hormone, CRH/CRF system mediates complex neuroendocrine functions such as responses to stress and energy balance via CRH receptor 1 (CRHR1) activation.^42^, ^43^, ^44^, ^45^ MRAP2 could interact with both MC4R and CRHR1 in vitro, but effect of MRAP2 on CRHR1 had not been extensively studied. Here we carried our intracerebroventricular (ICV) administration of CRF, the agonist of CRHR1 into MRAP2‐knockdown mice. Weight gain (Figure 8G) and 2 h‐food intake (Figure 8H) of ICV CRF mice significantly down‐regulated than that of controls. Overall, we demonstrated the in vivo evidence that the MRAP2–CRHR1 complex could regulates energy metabolism in the mouse hypothalamus. We speculated that each subset of neuronal cells uniquely marked by a GPCR or neuropeptide may function by forming a large complex with some co‐factors such as MRAP2, which together regulated energy homeostasis and might perform other complex physiological functions in vivo.

## 4 DISCUSSION

The MC4R, regulated by MRAP2, shows very extensive dimeric binding of numerous GPCR targets in the central nervous system.^46^ We have previously illustrated co‐localisation of MRAP2 with many other non‐melanocortin GPCRs, such as PKR1 and GHSR by analysing transcriptome data from hypothalamic cells.^47^ MRAP2 showed broad distribution in the mouse hypothalamus where it played a crucial role in the regulation of energy homeostasis. Furthermore, MRAP2 mutations (N88Y, L115V and R125C) identified in obese patients affect the ligand stimulation, trafficking and protein–protein interactions on major GPCR targets, indicating a potential regulatory role of these MRAP2 variants on other GPCR signalling cascades.^47^ Originally identified as MRAPs, the potential interactive network of MRAPs and regulations on other non‐melanocortin GPCRs have not been well explored.

Activation of the hypothalamic–pituitary–adrenocortical (HPA) axis can respond to acute threats to homeostasis or health state. An increase in circulating glucocorticoids mobilises energy and promotes some behavioural or physiological responses to stressors.^48^, ^49^ The energy balance of neuroendocrine circuits and systems under stress are largely overlapped. The pathways involved in regulating energy homeostasis also regulate the stress response.^50^, ^51^, ^52^ The neural circuits that control brain stress integration and systemic metabolism show significant functional coincidence. Consistent with this, metabolic disorders such as obesity and diabetes have a high co‐occurrence with stress‐related psychological diseases such as anxiety and depression.^53^, ^54^, ^55^

Here, we assessed the correlation coefficient of MRAP proteins and GPCRs in co‐expressing cells of the hypothalamus and adrenal gland, the two major endocrine organs for exerting the physiological functions of MRAP proteins (Figure 8A). We also integrated multiple human and mouse bulk and single‐cell RNA‐seq datasets to identify potential binding partners of MRAP proteins. With this approach, we found that various GPCR families showed very high correlation coefficients with MRAPs including orphan receptors and neuropeptide receptors, and these results were largely comparable across bulk and scRNA‐seq datasets (Figures 1, 2, 3).

The following co‐IP results showed obvious interactions between most sorted GPCRs and MRAPs (36 and 46 interacting partners of MRAP1 or MRAP2, respectively) (Figure 4). Furthermore, bimolecular fluorescence complementation assays clearly verified these interactions in live cells (Figure 5). All of these results strongly supported the anticipation from the bioinformatic analysis. We looked up the gene interaction network view of MRAP1/MRAP2 in the BioGRID database (Figures 8B and C) and found that most of these genes were not GPCRs and did not overlap with the interactors in this study (Figures 8D and E). This indicated that our study greatly bridged the gap of MRAP proteins in GPCR interaction network.

These findings spurred us to further assess the influence of MRAP proteins on GPCR signalling. We examined the pharmacological activity of four GPCRs interacted with MRAP proteins and related to metabolism: ADRB3, CRHR1, PTGER2 and PTGER4 with or without MRAPs in different concentrations of agonists or antagonists (Figure 6). MRAP1 and MRAP2 dose‐dependently inhibited the actions of these GPCRs. We expect to assess the whole pharmacological effects of MRAP2 on these screened GPCRs. We assumed that the constitutive activity changes may result in activation or loss of GPCR function, our results suggest that the constitutive activity changes of some GPCRs correlate with MRAP2 regulation of GPCR activity but some do not, suggesting that MRAP2 may regulate GPCR activity in other ways. Thus, we have further explored the effect of MRAP2 on membrane trafficking of GPCRs. Twenty GPCRs changed in membrane surface expression in the presence of MRAP1, and 33 GPCRs changed their surface expression in the presence of MRAP2. MRAP1 enhanced surface transport whereas MRAP2 reduced the trafficking of most GPCRs, suggesting a distinct role of MRAP1 and MRAP2 on modulating GPCR signal cascades (Figures 6, 7 and S7 and 8). The reduction of GPCRs surface expression might correspond to its signal suppression in the presence of MRAP2, like CRHR1 and PTGER2. However, the membrane surface expression of some GPCRs were elevated or unchanged, but their signalling activity was still reduced in the presence of MRAP2, such as ADRB3 and PTGER4. The effect of MRAP2 on GPCR function may also be due to a combination of other interacting factors, ion channels and altered physiological states.

Several prior studies have noted the vital role of MRAP2 on regulating non‐melanocortin GPCRs. For example, Srisai et al.^15^ observed that MRAP2 interacted with GHSR1a and promoted hunger sensing by potentiating ghrelin signalling. In addition, Chaly and Rouault found that the C‐terminal of MRAP2 could modulate the surface expression and inhibit the activity of PKR1 to control food intake whereas MRAP2 N‐terminus regulated OX1R trafficking and C‐terminus inhibited the activity.^12^, ^56^ These findings led to a new hypothesis and re‐definition that MRAP2 acted as a broad‐spectrum GPCR modulator. In vivo evidence supports the regulation of MRAP in energy homeostasis and metabolic health in adipose tissue.^57^ It has been suggested that MRAP may be associated with metabolically related GPCRs as well.^57^ Our study not only established a comparative and comprehensive in silico and biochemical approaches to explore novel GPCR partners of MRAP1 and MRAP2 in vitro but also identified several MRAP‐associated GPCRs involved in energy homeostasis (Tables 1 and 2).

Further in vivo evidence from animal studies, along with Genome Wide Association Studies (GWAS), pointed out that most of the identified novel GPCR partners interacting with MRAP1 or MRAP2 may have relevance to energy homeostasis and deficiency of which could lead to metabolic disorders and other neuronal related diseases (Table S1). Interestingly, we found that CRHR1 interacted with MRAP2 via Co‐IP and BiFC (Figures 4R and 5I), and the level of cAMP production of CRHR1 was inhibited by MRAP2 which may due to the reducing the membrane surface expression of CRHR1 (Figures 6F and 7G).

It is undeniable that some GPCRs are low expressed, but GPCRs act in a cascade effect and cells do not require much GPCR expression to function. In addition, at the single cell level only 3K–4K genes are generally detected, so many GPCRs are not visible at the mRNA level and may be overlooked. It must be stated that low GPCR expression may also give rise to important functions and these invisible GPCRs may functionally related to MRAP2. In summary, MRAP1 and MRAP2 are presented and redefined as broad‐spectrum GPCR modulators and act as vital physiological links to a large group of GPCRs in regulating energy homeostasis. Our findings could help us better understand the composite obese phenotype resulting from MRAP2 deficiency in vivo. The universal binding of MRAP2 to various GPCRs provides us new strategies for the development of novel transmembrane allosteric modulators for several pharmaceutically important GPCR targets.

## CONFLICT OF INTEREST

The authors declare that they have no competing interests.

## Supporting information

Supporting Information

Supporting Information
