# Supplementary material for: Identification of MRAP protein family as broad‐spectrum GPCR modulators
Source: Clin Transl Med. 2022 Oct 31;12(11):e1091. doi: 10.1002/ctm2.1091 (PMC9619224; doi:10.1002/ctm2.1091)

**Supplementary files**

**Figure S1 – Related to Figure 1. RNA and protein expression of human MRAPs from HPA database.**

(A) Protein tissue specificity of Mrap1 across 45 human tissues from the HPA database(Human Protein Atlas).

(B) RNA tissue specificity of Mrap1 across 45 human tissues from the HPA database.

(C) RNA tissue specificity of Mrap2 across 45 human tissues from the HPA database.

(D) Gene oncology treemap of Mrap1 in the integrative single cell RNA-seq datasets of the human adrenal gland.

(E) Gene oncology treemap of Mrap2 in the integrative single cell RNA-seq datasets of the human hypothalamus.

**
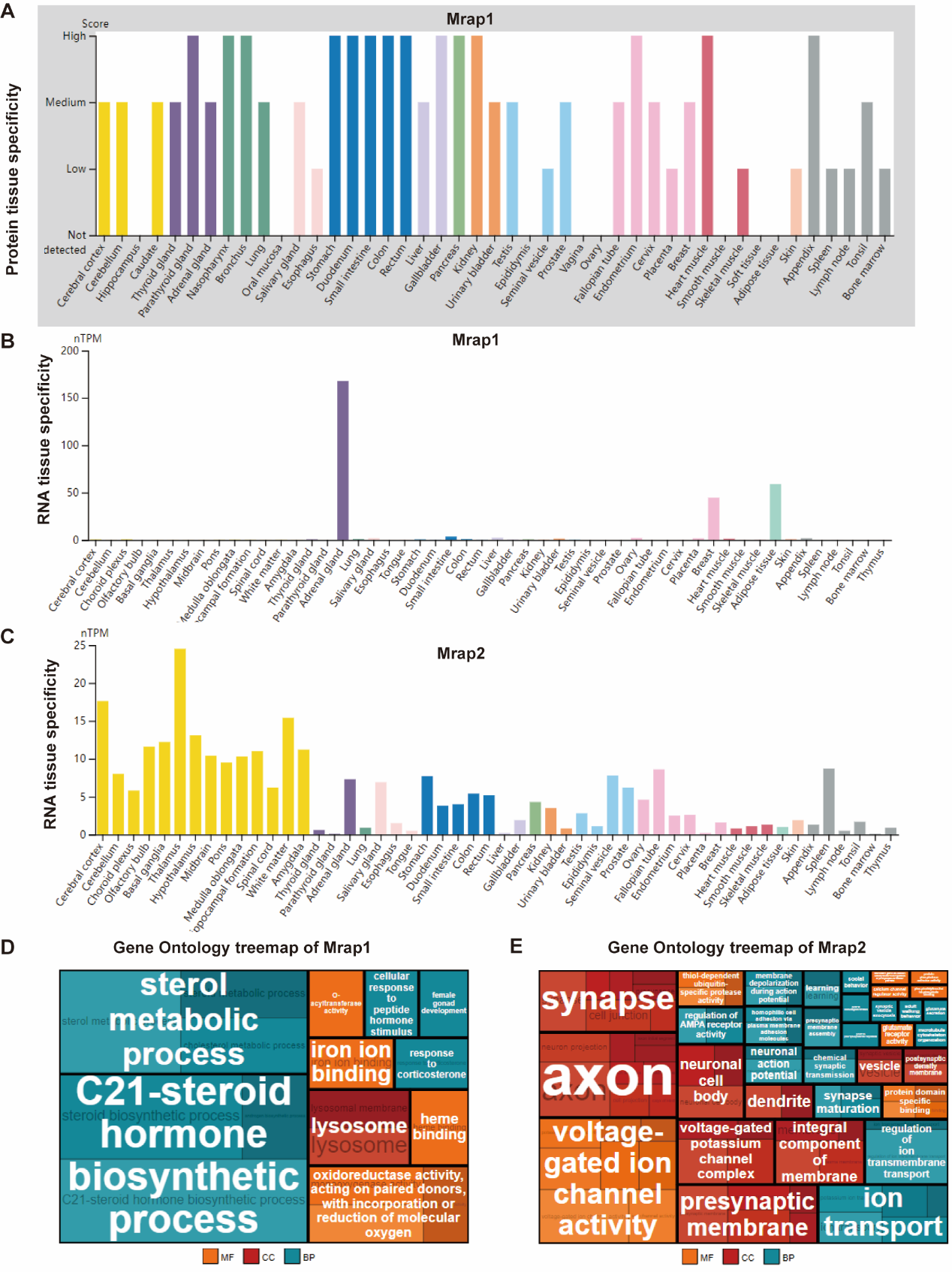
**

**Figure S2 – Related to Figure 2. Expression of GPCR and MRAPs in human and mouse hypothalamic single-cell public database.**

(A) Heatmap of the differentially expressed GPCRs in 3 neural cell types of the human hypothalamus.

(B) Violin plots of the expression profile of cell marker genes and MRAP2 across 3 neural cell types in the human hypothalamus.

(C) tSNE plot identification of 3 neural cell types in the integrative single cell RNA-seq datasets of the human hypothalamus.

(D) tSNE plot identification of 10 cell types in the integrative single cell RNA-seq datasets of the mouse hypothalamus.

(E) Dot plot of marker genes in different cell types of mouse hypothalamus.

(F) Expression of MRAP1 and MRAP2 in all cell types of the mouse hypothalamus.

(G) Heatmap of the differentially expressed GPCRs in the mouse hypothalamus.


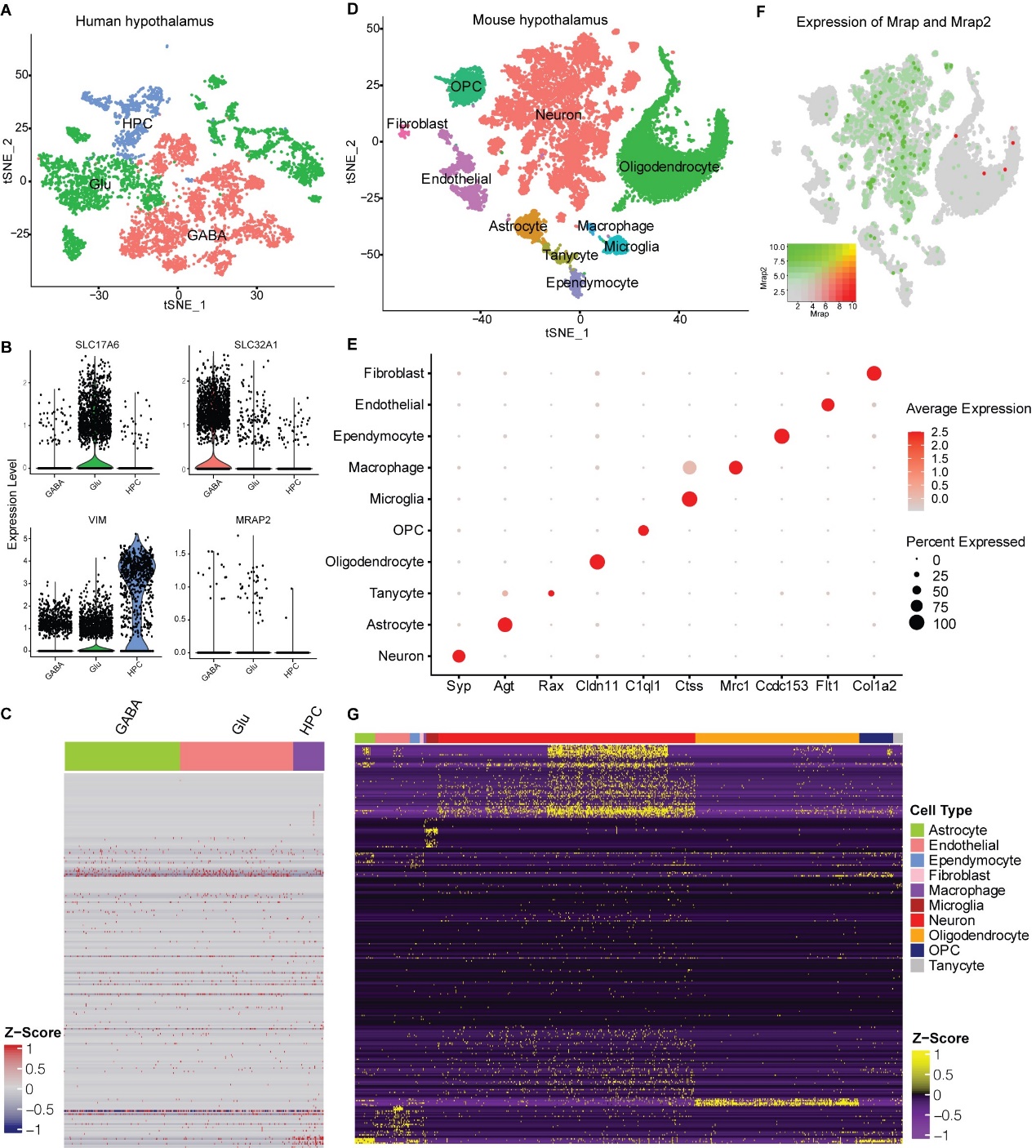


**Figure S3 – Related to Figure 3. The co-expression network of MRAP1 and GPCRs in single-cell datasets of mouse adrenal gland.**

(A) tSNE plot of 10 cell types identified in the integrative single cell RNA-seq datasets of the mouse adrenal gland.

(B) Dot plot of classic marker genes used to identify cell identities in mouse adrenal gland.

(C) Expression of MRAPs in different cell types of the mouse adrenal gland.

(D) Expressions of all GPCRs and MRAP1 across cell types of mouse adrenal gland.

(E) Expression profiles of the detected GPCRs across cell types of mouse adrenal gland.

**
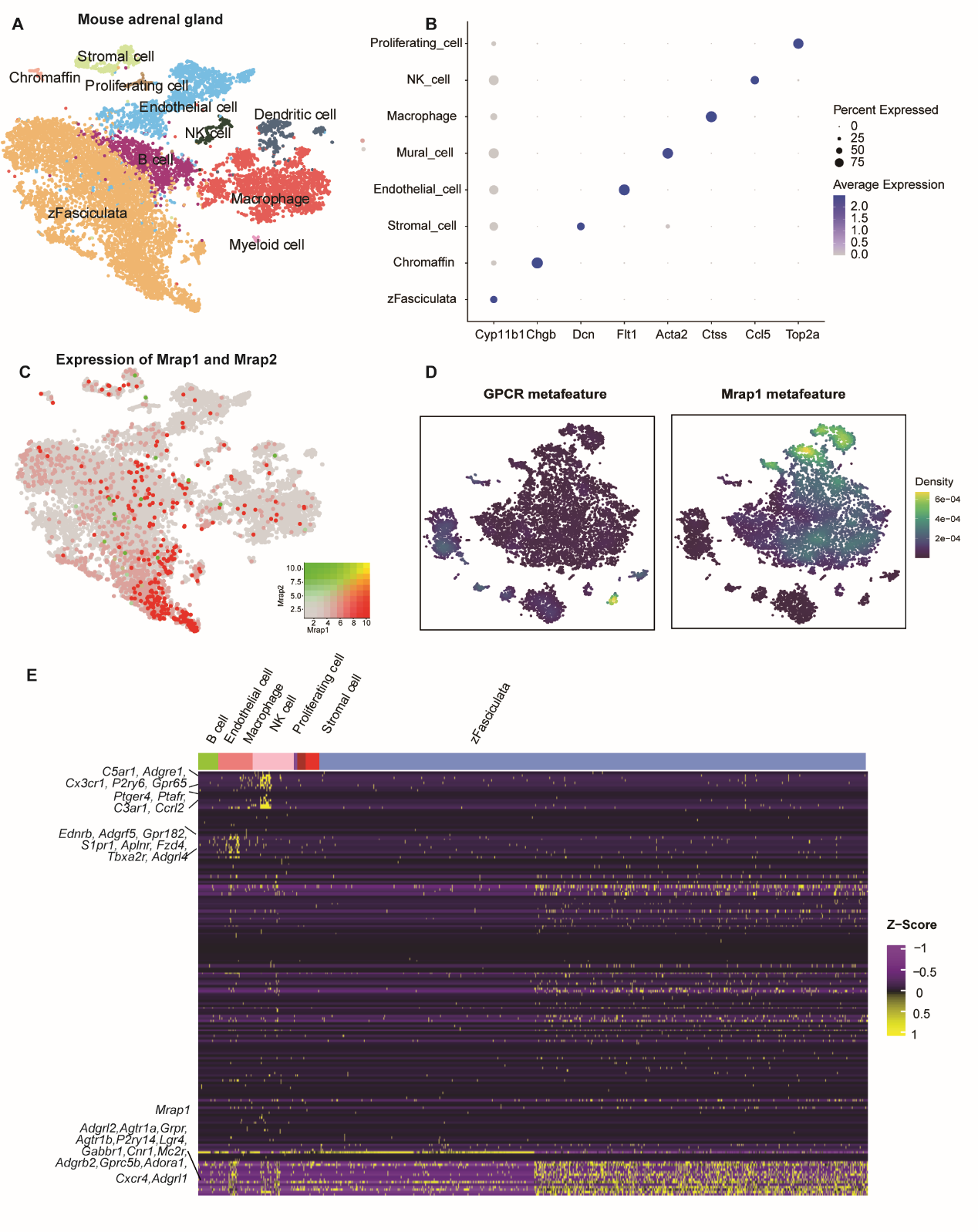
**

**Figure S4 – Related to Figure 4. GPCRs interact with MRAPs.**

Positive co-immunoprecipitation results of 2Flag-MRAP1(A) or 2Flag-MRAP2(B) with selected GPCRs. GPCRs were immunoprecipitated using rabbit anti-HA antibody (IP part on the left) and detected using mouse anti-HA antibody (upper membrane). MRAP1 and MRAP2 were detected using mouse anti-Flag antibody (lower membrane). For a Western blot control, one tenth of the lysate was used. No antibody was used as a control for the IP (lysate part on the right).


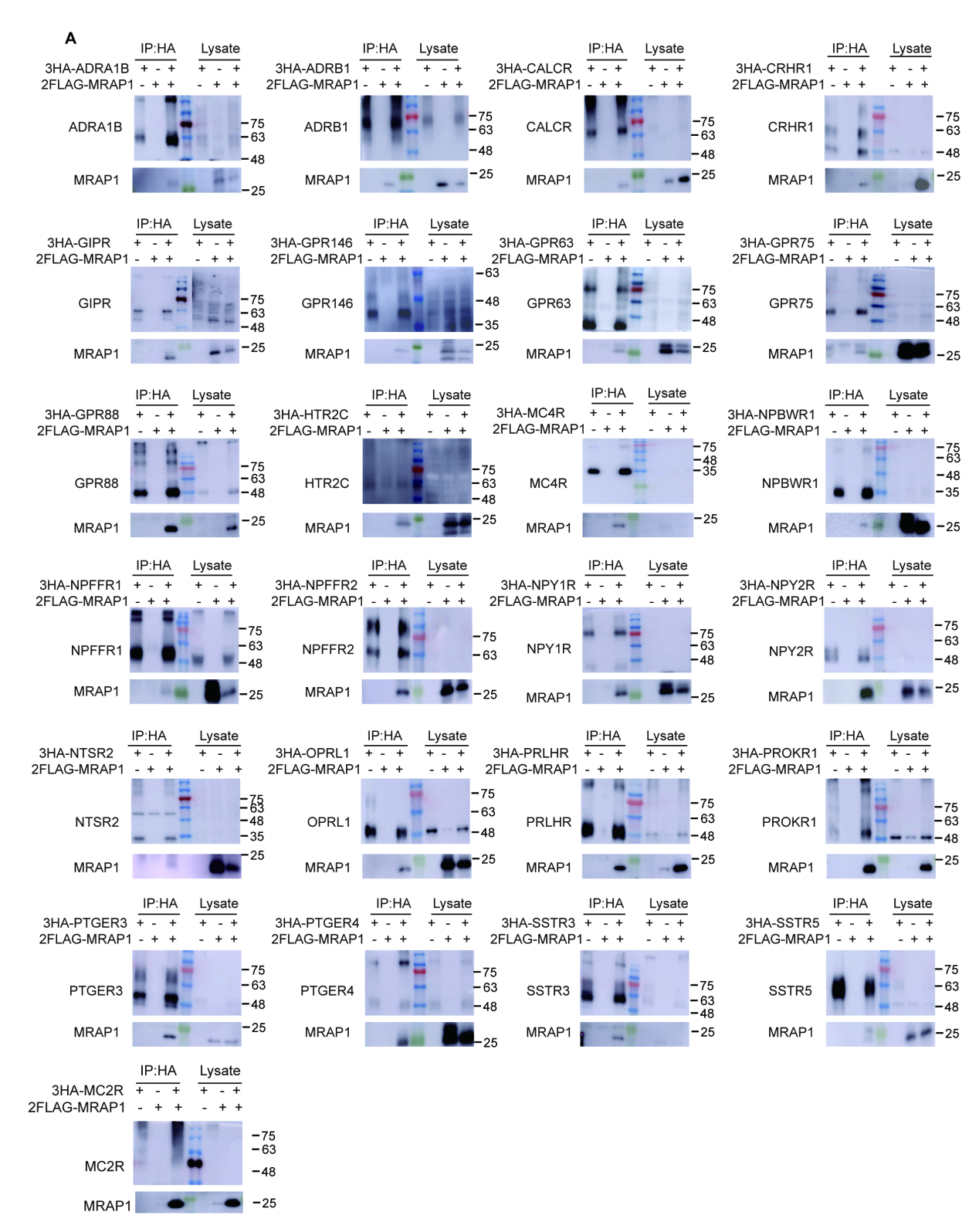


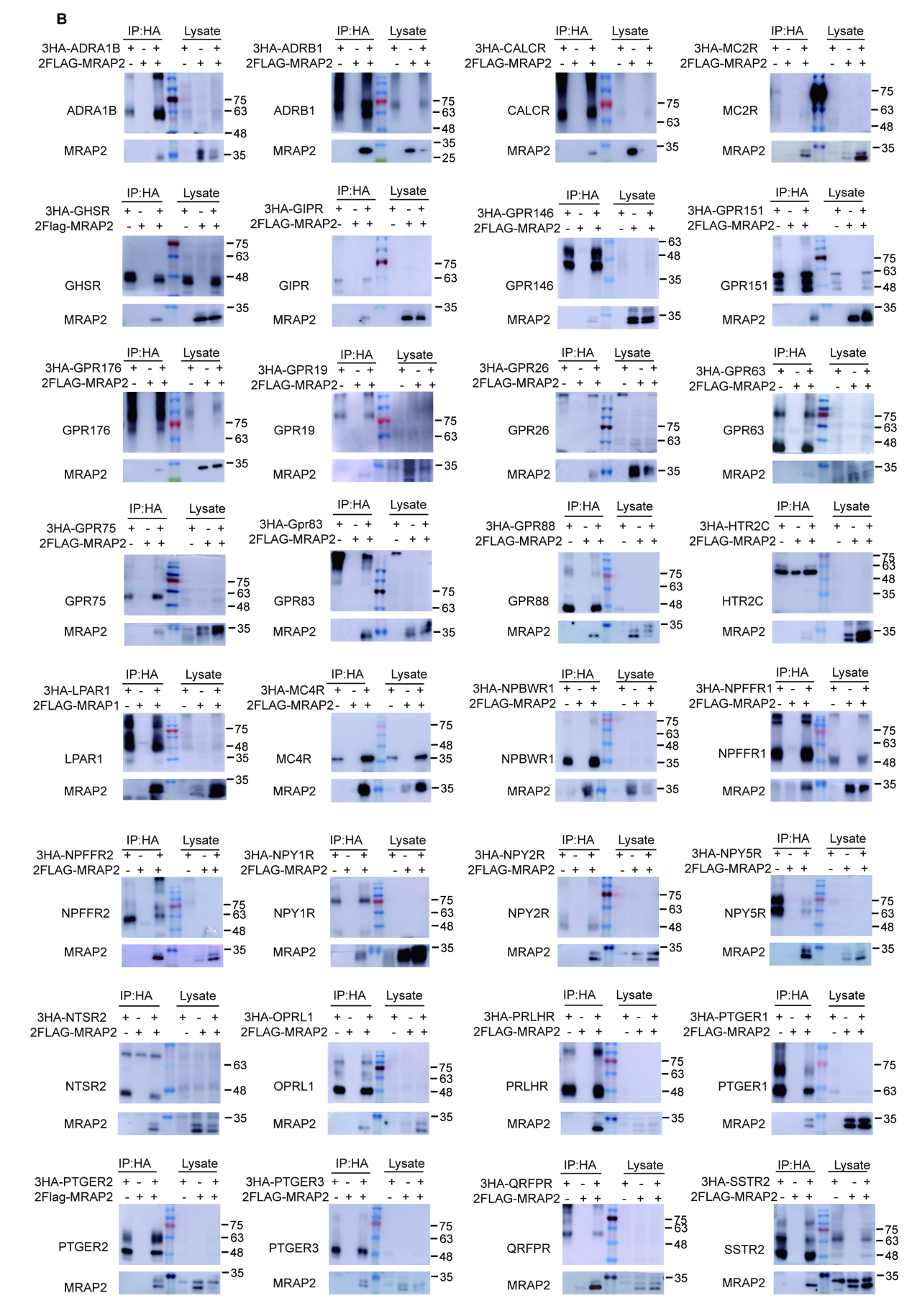


**Figure S5 – Related to Figure 4. GPCRs without interactions with MRAPs.**

Negative co-immunoprecipitation results of 2Flag-MRAP1(A) or 2Flag-MRAP2(B) with selected GPCRs. GPCRs were immunoprecipitated using rabbit anti-HA antibody (IP part on the left) and detected using mouse anti-HA antibody (upper membrane). MRAP1 and MRAP2 were detected using mouse anti-Flag antibody (lower membrane). For a Western blot control, one tenth of the lysate was used. No antibody was used as a control for the IP (lysate part on the right).


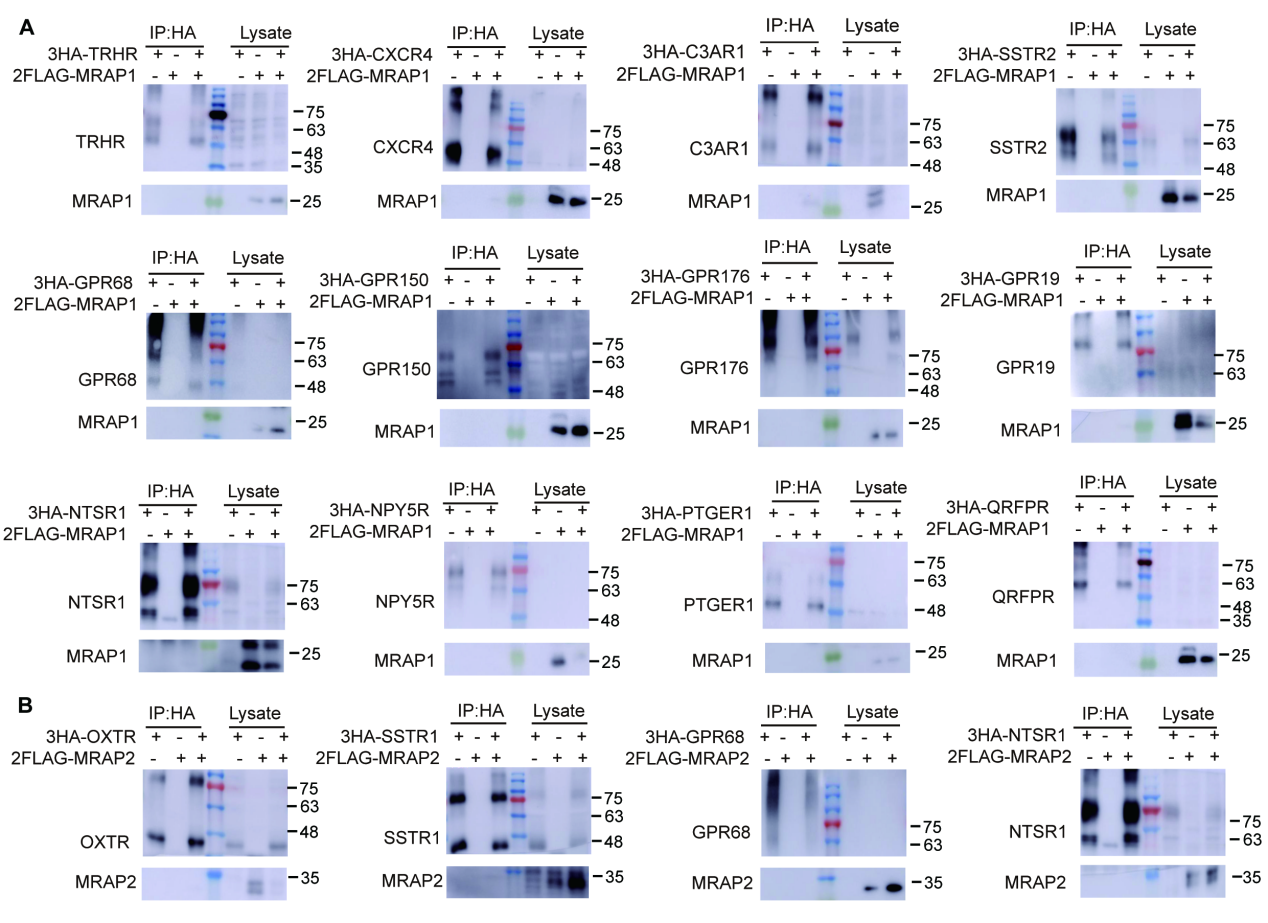


**Figure S6-** **Related to Figure 5. Co-localization of selected GPCRs and MRAP proteins in cells.**

Selected GPCRs were co-transfected with MRAP1-Flag-F2(A) or MRAP2-Flag-F2(B). Nuclei stained with DAPI are shown in blue and YFP fluorescence in yellow. MRAP1 and MRAP2 emitted red fluorescence after incubation with Flag antibody and Alexa Fluor594 secondary antibody. Each YFP group was repeated at least twice, with three fields of view selected.

**
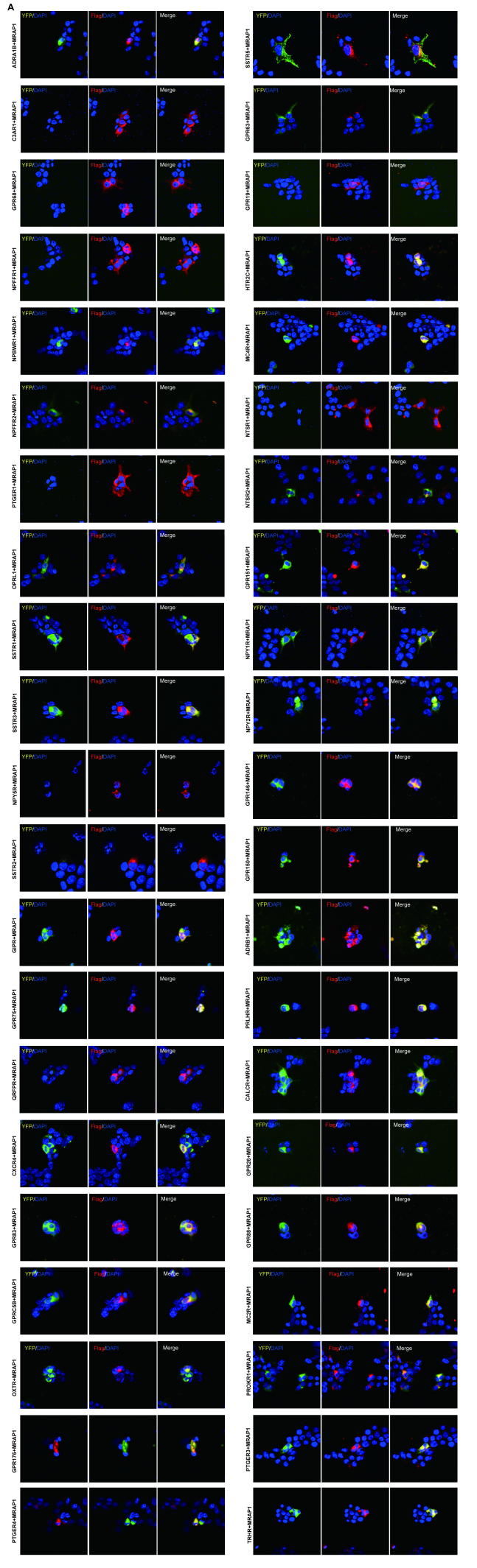

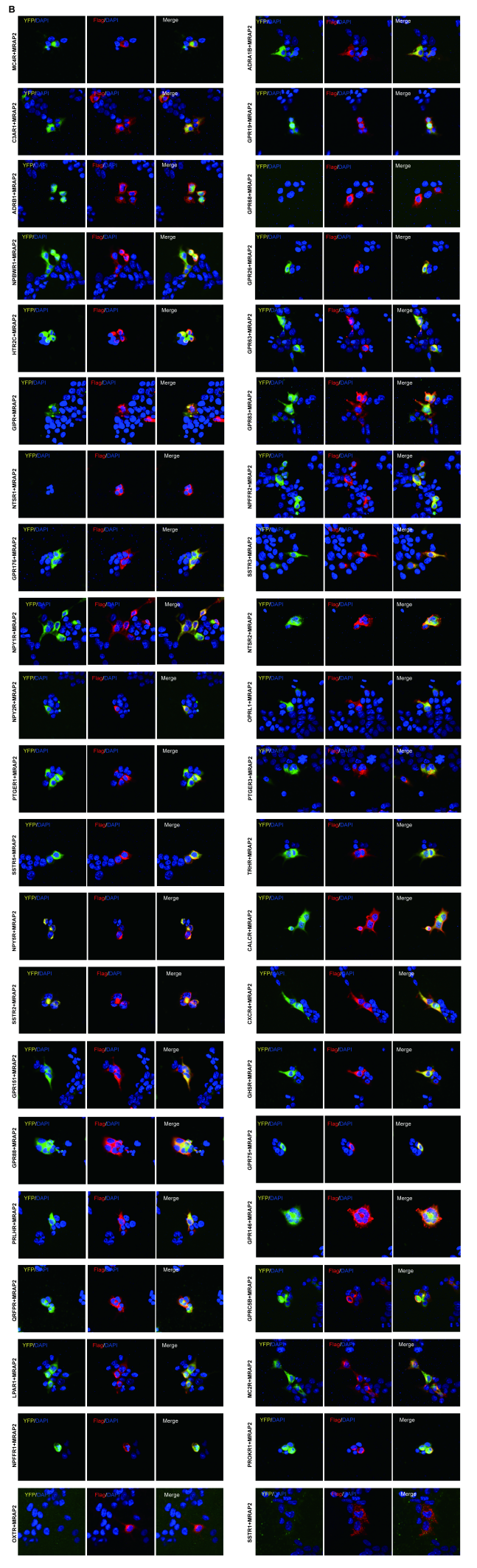
**

**Figure S7-** **Related to Figure 6. Modulation of the constitutive activities of GPCRs by MRAPs.**

Measurement of the constitutive activities of selected GPCRs in the presence of various doses of MRAP1 or MRAP2 proteins.


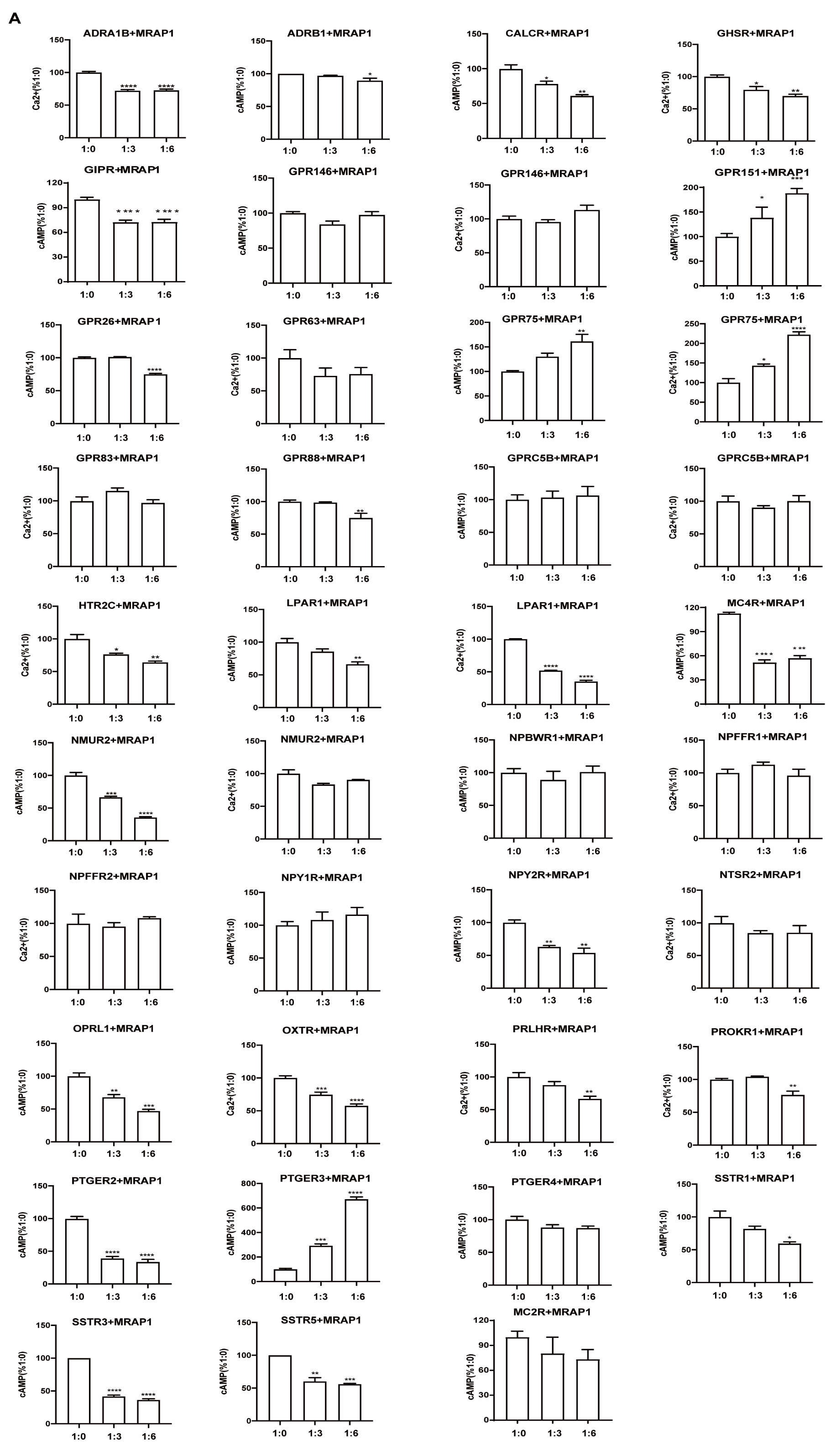


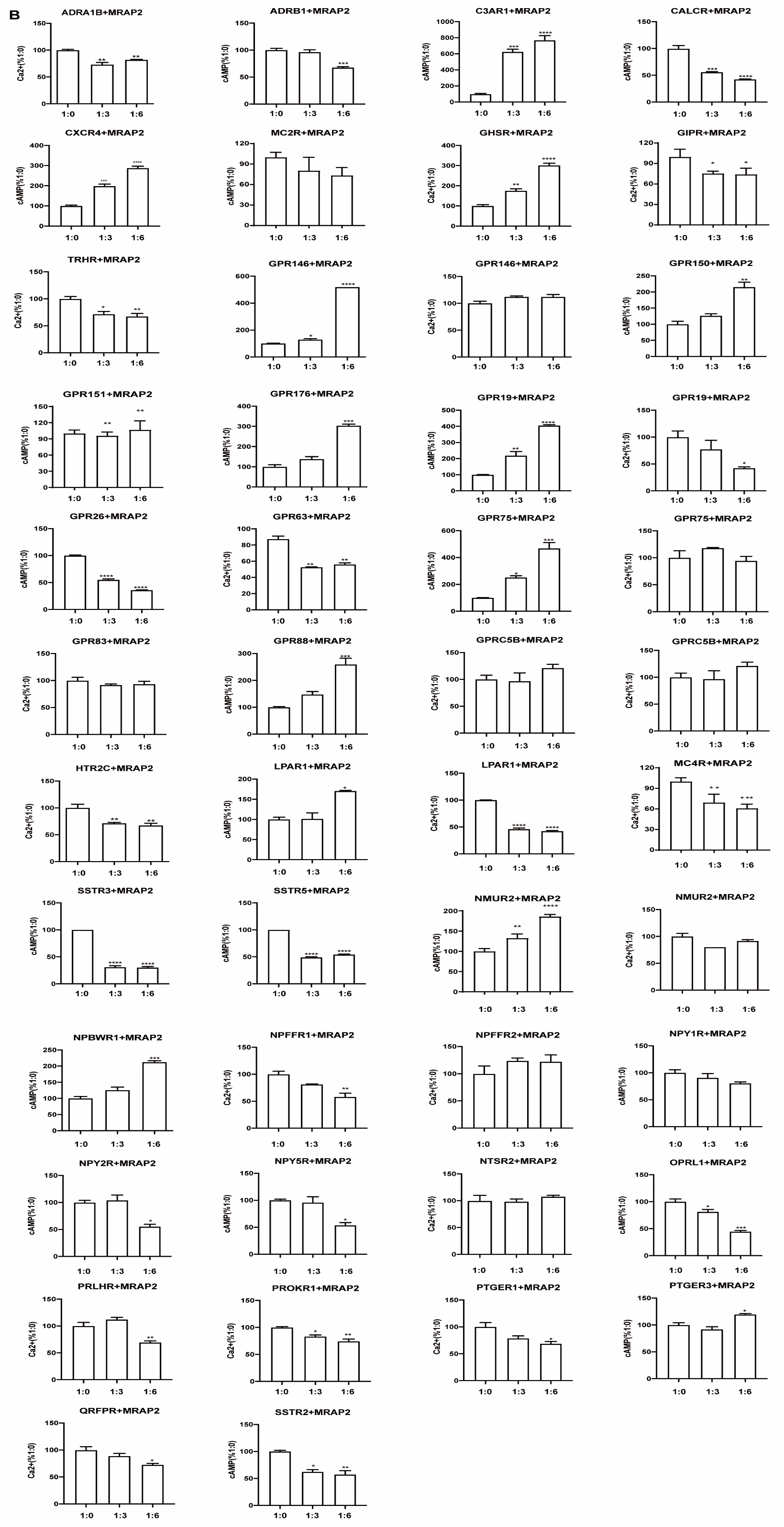


**Figure S8 – Related to Figure 7. Surface expression of GPCRs with or without MRAPs.**

Surface expression of selected GPCRs in HEK293 cells transfected with an empty vector or MRAP1 or MRAP2 at 1:3 and 1:6 ratio using cell ELISA assays. One-way ANOVA with post hoc Tukey. ns (no significant change), *p<0.05, **p<0.01, ****p<0.001, ****p<0.0001.


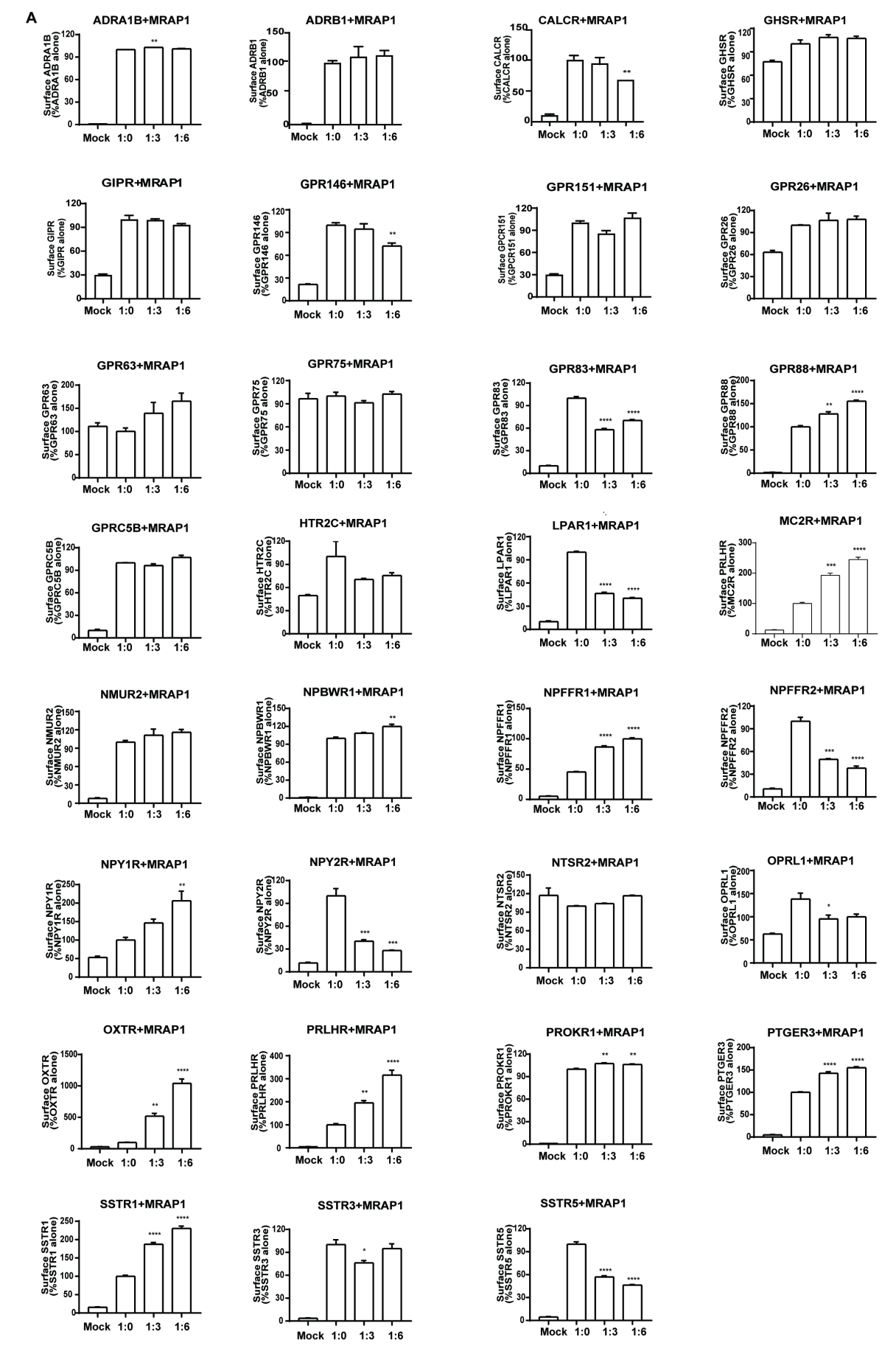


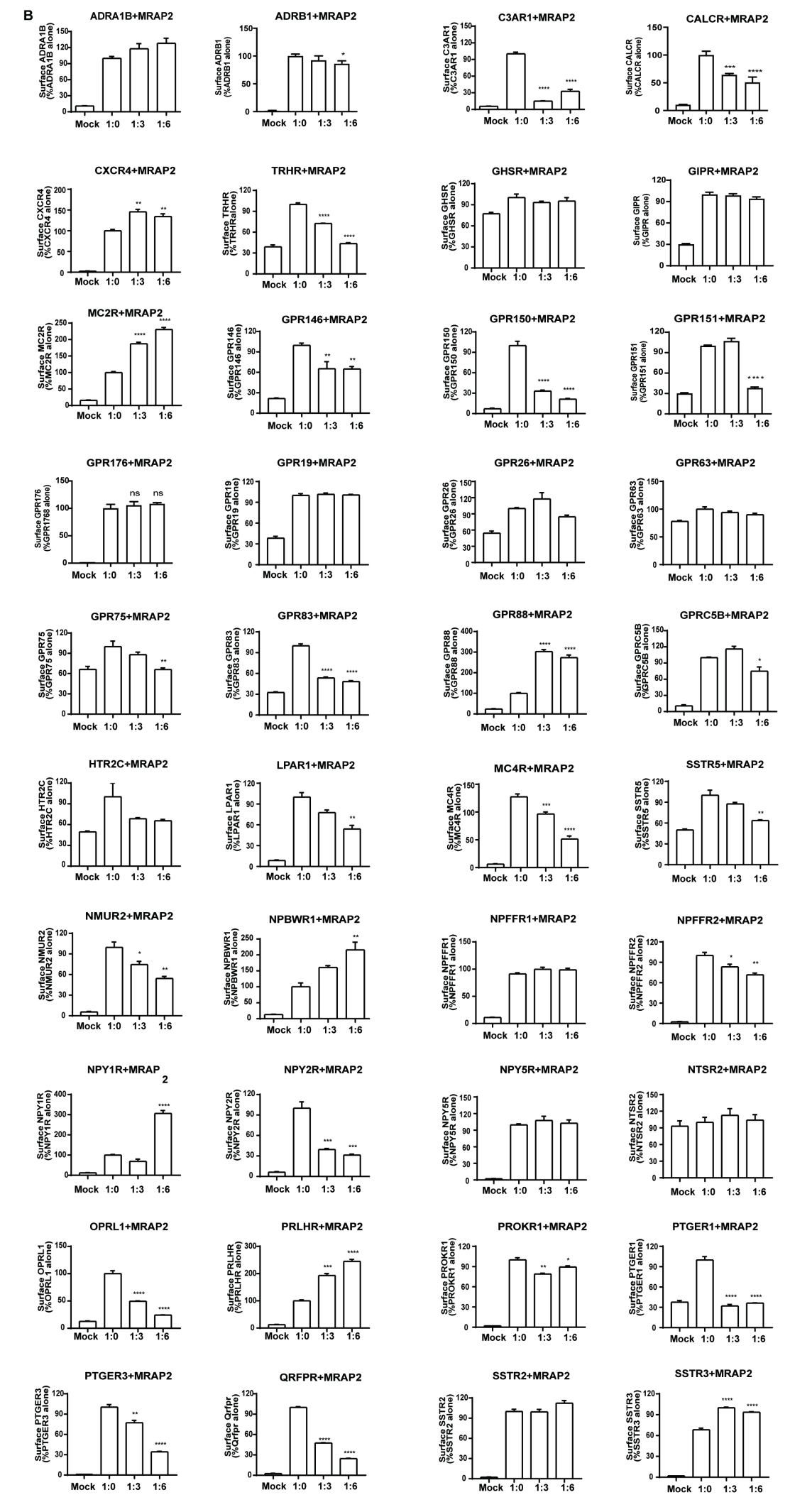

Supplement: Supplementary file 1 — Supporting Information [file CTM2-12-e1091-s002.docx]
