# Supplementary material for: Identification of MRAP protein family as broad‐spectrum GPCR modulators
Source: Clin Transl Med. 2022 Oct 31;12(11):e1091. doi: 10.1002/ctm2.1091 (PMC9619224; doi:10.1002/ctm2.1091)
Supplement: Supplementary file 2 — Supporting Information [file CTM2-12-e1091-s001.docx]

**Table 3:** Statistical analysis of agonist stimulated GPCRs activity in the presence of different doses of MRAP2.

| **Data statistics of Fig.7A-7H** | | **LogEC50** | | | **P value for Vmax comparison** | | |
| --- | --- | --- | --- | --- | --- | --- | --- |
|  |  | **1:0** | **1:3** | **1:6** | **1:0 vs. 1:3** | **1:0 vs. 1:6** | **1:3 vs. 1:6** |
| Fig.7A | ADRB3:MRAP1 | -9.98±0.06 | -10.30±0.11 | -10.39±0.19 | 0.0654 | 0.0149 | <0.0001 |
| Fig.7B | CRHR1:MRAP1 | -9.88±0.06 | -9.77±0.08 | -9.42±0.17 | 0.0011 | 0.0104 | 0.2564 |
| Fig.7C | PTGER2:MRAP1 | 9.92±0.14 | -9.70±0.17 | -9.51±0.22 | <0.0001 | <0.0001 | 0.0002 |
| Fig.7D | PTGER4:MRAP1 | -11.36±0.80 | -10.87±0.39 | -10.23±0.66 | <0.0001 | <0.0001 | 0.0018 |
| Fig.7E | ADRB3:MRAP2 | -9.90±0.08 | -9.87±0.07 | -9.82±0.04 | <0.0001 | <0.0001 | 0.3960 |
| Fig.7F | CRHR1:MRAP2 | -10.68±0.11 | -10.62±0.09 | -10.54±0.10 | <0.0001 | <0.0001 | <0.0001 |
| Fig.7G | PTGER2:MRAP2 | -9.92±0.14 | -9.65±0.15 | -9.48±0.13 | <0.0001 | <0.0001 | <0.0001 |
| Fig.7H | PTGER4:MRAP2 | -11.36±0.80 | -10.95±0.36 | -10.84±0.45 | <0.0001 | <0.0001 | 0.0139 |

Values were expressed as the mean ± S.E.M. of at least three independent experiments. Two-way ANOVA with Tukey post-test was applied in the statistical analysis.
